# Dysregulated *HELLS* expression alters cellular processes and serves as a potential prognostic marker in acute myeloid leukemia

**DOI:** 10.1016/j.jbc.2026.113210

**Published:** 2026-05-28

**Authors:** Swati Madhulika, T Sayamsmruti Panda, Priyanka Samal, Sreelakshmi S. Kumar, Smrutishree Mohanty, Monalisa Ghosh, Sohini Chakraborty, Asima Das, Jyochnamayi Panda, Subha Saha, Mrutyunjaya Padhy, Tareni Prasad Mallick, Preeti Pranjya Mohapatra, Punit Prasad

**Affiliations:** 1 Epigenetics and Chromatin Biology Unit, Biotechnology Research and Innovation Council (BRIC)- Institute of Life Sciences, NALCO Square, Bhubaneswar, Odisha, India; 2 Regional Centre for Biotechnology, Faridabad, Haryana, India; 3 Department of Clinical Hematology and Stem Cell Transplant, IMS & SUM Hospital & Medical College, Bhubaneswar, Odisha, India; 4 Department of Pathology, New York University Grossman School of Medicine, New York, New York, USA; 5 Department of Obstetrics and Gynaecology, Kalinga Institute of Medical Sciences Hospital, Bhubaneswar, Odisha, India; 6 Krantz Family Center for Cancer Research, Massachusetts General Hospital, Boston, Massachusetts, USA; 7 Harvard Medical School, Boston, Massachusetts, USA; 8 Broad Institute of the Massachusetts Institute of Technology and Harvard University, Cambridge, Massachusetts, USA

**Keywords:** HELLS, AML, epigenetics, differentiation, apoptosis

## Abstract

Spatiotemporal gene expression is regulated by the SNF2 family of ATPases that remodel chromatin, among which helicase lymphoid-specific (*HELLS*) play an essential role in DNA-templated processes. Analysis from cancer databases revealed *HELLS* upregulation in several cancers, including acute myeloid leukemia (AML). Using an *in vitro* myeloid differentiation model, we found that HELLS deficiency promotes myeloid differentiation, apoptosis, cell-cycle arrest, and chromatin instability. Furthermore, HELLS deficiency enhanced myeloid differentiation and apoptosis in HL-60 cells when treated with chemotherapy drugs, including 5-azacytidine, cytarabine, and doxorubicin. Epigenetically, loss of HELLS reduced active histone mark (H3K4me3) and enhanced repressive promoter (H3K27me3), active enhancer (H3K27ac) marks, and chromatin accessibility. Transcriptomic analysis of BeatAML data and bioinformatics analysis revealed that *HELLS* upregulation is associated with adverse prognosis, as defined by the ELN-2017 classification, and significantly poorer survival. Classification of AML patients into HELLS^High^ and HELLS^Low^ gene-expression groups showed that lower *HELLS* expression was associated with a favorable prognosis and improved remission. HELLS upregulation was positively and negatively associated with leukemic stem/progenitor cell (LSPC) markers and myeloid lineage markers, respectively. *Ex vivo* validation in AML patient-derived LSPCs demonstrated that HELLS deficiency reduces leukemic marker burden (CD123 and TIM-3) and increases apoptosis. Overall, we show HELLS upregulation drives leukemogenesis with poor outcomes, highlighting its potential as a prognostic marker and therapeutic target in AML.

Helicase, lymphoid-specific (*HELLS*), also known as *LSH*, *SMARCA6*, and *PASG*, is a SNF2 family of chromatin remodelers, which was first identified in murine thymus (1, 2). HELLS expression is predominantly observed in proliferating tissues such as the thymus, bone marrow, and testis, whereas it is absent in nonproliferative tissues of the brain, heart, liver, and kidney. This highlights its critical role in regulating cellular proliferation across diverse cell lineages (2, 3). HELLS is associated with diverse DNA template-dependent processes, including DNA repair, recombination, methylation, cell cycle, and stem cell proliferation (4, 5, 6). Further, HELLS deficiency promotes genome instability by multiple mechanisms, such as sensitization of DNA to DNA-damaging agents, delay in DNA double-strand break repair (7), higher frequency of replication stress, chromosomal aberrations, fragile telomeres, stalling replication fork (8, 9), compromising the canonical nonhomologous end joining pathway, and delaying XRCC5 accumulation at DNA damage sites (10). These mechanisms lead to enhanced apoptosis, aneuploidy, and abnormal chromosomal segregation.

The HELLS regulate nucleosome positioning, histone variant incorporation, and chromatin accessibility during transcription. It promotes incorporation of macroH2A, leading to transcriptional repression at repetitive regions and developmental enhancers (8, 11, 12, 13). The nucleosome sliding activity of HELLS exposes CpG sites for DNA methylation and contributes to heterochromatin formation, a process dependent on CDCA7 (8). Beyond gene repression, HELLS can also promote transcription by generating nucleosome-depleted regions, which facilitate the binding of transcription factors and RNA polymerase (13).

The precise regulation of *HELLS* expression is crucial for normal development, cellular proliferation, and the prevention of epigenetic aberrations, which can lead to its overexpression and subsequent malignant phenotypes (4). Upregulation of *HELLS* in several cancers, including pancreatic, hepatocellular, colorectal, lung, head and neck, and cervical cancers, glioblastoma, retinoblastoma, osteosarcoma, medulloblastoma, and lymphomas, confers a proliferative advantage and the ability to resist apoptosis in malignant cells, thereby promoting malignancies (4, 14). Multiple studies have shown that upregulation of HELLS leads to abnormal cellular proliferation and alterations in genome stability, DNA repair mechanisms, signaling pathways, and epigenetic pathways, thereby promoting tumorigenesis (4, 14). Several noncoding RNAs, including circular RNAs (circ-RNAs), and other proteins, such as FOXM1, E2F1, E2F3, and LMP1, are key factors in maintaining high HELLS expression in various cancers (15, 16, 17, 18, 19, 20, 21).

Previous reports demonstrated that loss of HELLS was associated with impaired B-cell development and immunoglobulin class-switch recombination (22, 23). Mice transplanted with Lsh^−/−^ hematopoietic precursor cells from fetal liver accounted for a reduced survival rate and significant reduction in erythrocyte number, hematocrit, and leukocyte count (24). In addition, a comprehensive bioinformatics analysis of cancer databases revealed *HELLS* upregulation in several solid tumors and myeloid malignancies, acute myeloid leukemia (AML) (14), and suggested *HELLS* as a potential prognostic marker, and T-cell/B-cell acute lymphoblastic leukemia (25). In a separate study, a 75-nucleotide in-frame deletion in exon 18 was found in a significant number of patients with leukemia but not in normal bone marrow cells, suggesting its association with disrupted normal hematopoietic cell functions (26).

The hyper-proliferative nature of leukemic blasts and relapses due to survival of leukemic stem cells (LSCs) poses a significant threat to the survival of patients with AML. Although there is an indicative study that upregulation of *HELLS* may lead to AML aggressiveness, there is no descriptive study to identify the role of HELLS in myeloid malignancy. This prompted us to conduct this study, which shows that *HELLS* upregulation in AML plays a significant role in leukemic cell proliferation. HELLS deficiency resulted in enhanced myeloid differentiation, apoptosis, cell cycle arrest, and enhanced chemotherapeutic drug efficacy. Comprehensive bioinformatic analysis and *ex vivo* validation further demonstrated that HELLS depletion reduces leukemic burden. Our data strongly suggest that upregulation of *HELLS* is a key factor that alters the hematopoietic landscape, promotes AML pathology, and is therefore a potential prognostic biomarker.

## Results

### Expression of *HELLS* in hematopoietic stem/progenitor cells is significantly reduced upon myeloid differentiation

HELLS is essential and highly expressed in undifferentiated embryonic stem cells, neuronal and hematopoietic stem/progenitor cells, and proliferating cells such as lymphoid cells/tissue, and germ cells (2, 26, 27, 28). However, its expression is reduced in terminally differentiated cells, indicating its role in cellular proliferation. To investigate *HELLS* expression in normal hematopoiesis across lineage-committed hematopoietic cells, we first mined the “BLOODSPOT” database. *HELLS* expression is upregulated in hematopoietic stem cells (HSC), multipotential progenitors, common myeloid progenitors (CMP), granulocyte-monocyte progenitors, and myelocytes. As hematopoietic cells commit to myeloid differentiation, *HELLS* expression significantly decreases, as observed in meta-myelocytes, band cells, and monocytes (Mono) (Fig. S1*A*). A previous report shows high *HELLS* expression in hematopoietic progenitors and significantly lower expression in monocytes and granulocytes (26). Similar results were obtained from a second dataset, GSE74246, in which *HELLS* expression is high in HSCs, common myeloid progenitors, and granulocyte-monocyte progenitors and low in differentiated monocytes. The results of the promoter accessibility assay further confirmed that the *HELLS* promoter is regulated, with its expression modulated across different hematopoietic cell types (Fig. S1, *B* and *C*). Finally, we validated *HELLS* expression using *ex vivo* cord blood-derived CD34^+^ hematopoietic stem and progenitor cells (HSPCs) and *in vitro* myeloid differentiation models (29). *HELLS* expression in CD34^+^ HSPCs was significantly reduced in granulocyte colony-stimulating factor and macrophage colony-stimulating factor (M-CSF)-differentiated myeloid cells (Fig. S1*D*). Thus, *HELLS* expression is upregulated in proliferating cells in normal hematopoietic systems.

### *HELLS* is upregulated across cancers, including AML

**Figure 1:**
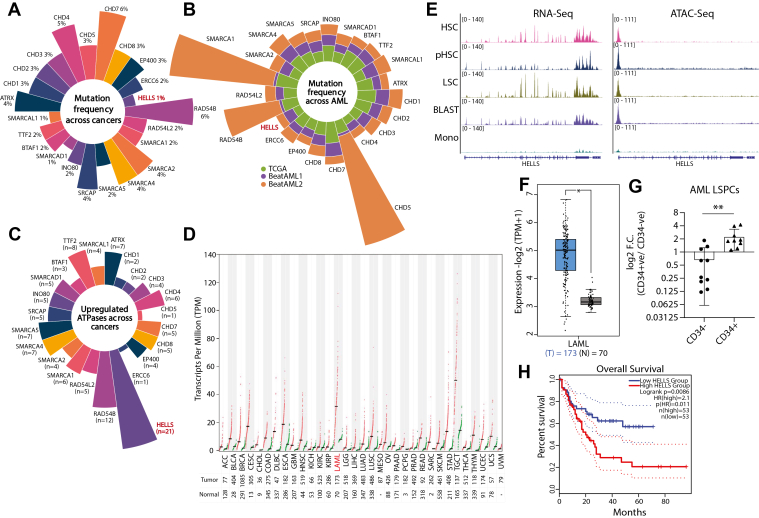
***HELLS* gene expression is upregulated across cancers, including AML.***A*, polar charts showing mutation frequency of several chromatin remodeler ATPases curated from the cBioportal (https://www.cbioportal.org/) database. *B*, mutation frequency of chromatin remodeler ATPases across different AML datasets, TCGA (firehose), BeatAM1.0 (Nature, 2018), BeatAML2.0 (Cancer, 2022). *C*, upregulated gene expression of several ATPases across different cancers curated from GEPIA2 database (http://gepia2.cancer-pku.cn/#index). *D*, gene expression of *HELLS* across several types of cancer curated from GEPIA2. *E*, genome browser plot of RNA-sequencing peaks at gene bodies of *HELLS* across AML cells HSC (n = 04), patient-HSC (p-HSC) (n = 04), leukemic stem cells (LSC) (n = 07), blast cells ((blast low (n = 05) and blast high (n = 06)), and monocytes (n = 04), respectively, from GSE74246 (*left panel*). Genome browser plot of ATAC-sequencing peaks at *HELLS* promoter region across AML cells HSC (n = 07), patient-HSC (p-HSC; n = 19), leukemic stem cells (LSC; n = 08), blast cells (n = 13), and monocytes (n = 06), respectively, from GSE74912 (*right panel*). *F*, gene expression of *HELLS* across AML patients (n = 173) and normal (n = 70) healthy individual samples curated from GEPIA2 database. *G*, relative log2 fold change of *HELLS* in CD34+ AML cells (n = 9) compared with that in the CD34-negative population (n = 10). *H*, Kaplan-Meier curve of overall survival for AML patients from the GEPIA2 database (n = 53). Statistical parameters in this figure are unpaired *t* test, ∗ *p*-value < 0.05 ∗∗ *p*-value < 0.01. HELLS, helicase lymphoid-specific; AML, acute myeloid leukemia; TCGA, The Cancer Genome Atlas; HSC, hematopoietic stem cell.

Genetic lesions in oncogenes, tumor suppressor genes, and DNA repair genes disrupt normal gene regulatory mechanisms, resulting in unregulated cell proliferation. Further, upregulation of normal or mutant genes can also result in a malignant phenotype. We were interested in understanding whether any chromatin-remodeling ATPases are upregulated in AML and play a role in promoting leukemogenesis. Therefore, to identify genetic aberrations and their potential role in malignancies, we curated mutation frequencies within 15 chromatin remodeling ATPases across different cancers using The Cancer Genome Atlas (TCGA) database where mutation frequencies were found to be low, ranging from 1 to 6% (Fig. 1*A*). For AML samples, we curated mutation frequencies of chromatin remodeling ATPases from TCGA, BeatAML1.0, and BeatAML2.0 data sets using cBioportal (Fig. 1*B*) (30, 31, 32). The mutation frequencies for most of the ATPases were consistent in three datasets except for *CHD5*, *SMARCA1*, and *RAD54B*, which showed very high mutation frequencies of 42%, 39%, and 21%, respectively in the BeatAML2.0 data sets (Fig. 1*B*). Interestingly, *HELLS* showed a considerably low mutation frequency of 1% and was highly upregulated in several cancers (n = 21) (Fig. 1*C*). Further, the median gene expression data for *HELLS* shows upregulation in most of the cancers, including AML, compared with that in normal tissues (Fig. 1*D*). Next, we curated transcriptomic data from GSE74246 and compared *HELLS* expression across HSCs, patient HSCs, LSCs, and blasts from AML patients, and monocytes. The read count density was higher in LSCs than in HSCs, patient HSCs, and leukemic blasts. Differentiated monocytes showed very low *HELLS* expression, suggesting a role in cell proliferation and stem cell maintenance (Fig. 1*E*, left panel). To further understand whether *HELLS* expression was regulated epigenetically, we curated ATAC-seq data (GSE74912) for the same hematopoietic lineages and found that promoter accessibility was significantly reduced in monocytes compared with that in other cell types, suggesting that *HELLS* expression is directly regulated *via* upstream element regulation (Fig. 1*E*, right panel). Curated gene expression data from GEPIA2 further confirms significant upregulation of *HELLS* in patients with AML (Fig. 1*F*). Finally, we used an *ex vivo* model to determine *HELLS* expression in CD34^+^ AML patient-derived leukemic stem/progenitor cells (LSPCs) and compared it with expression in CD34^-^ cells from the same samples. We observed *HELLS* upregulation in the CD34^+^ fraction in patient-derived CD34^+^ LSPCs (Fig. 1*G*). TCGA data further demonstrated that *HELLS* upregulation in AML patients is associated with a higher hazard ratio (HR) and poorer overall survival (OS) rate (Fig. 1*H*). Taken together, these data demonstrate that *HELLS* expression is upregulated in patients with AML, with a poorer OS rate.

### Loss of *HELLS* promotes myeloid differentiation

**Figure 2:**
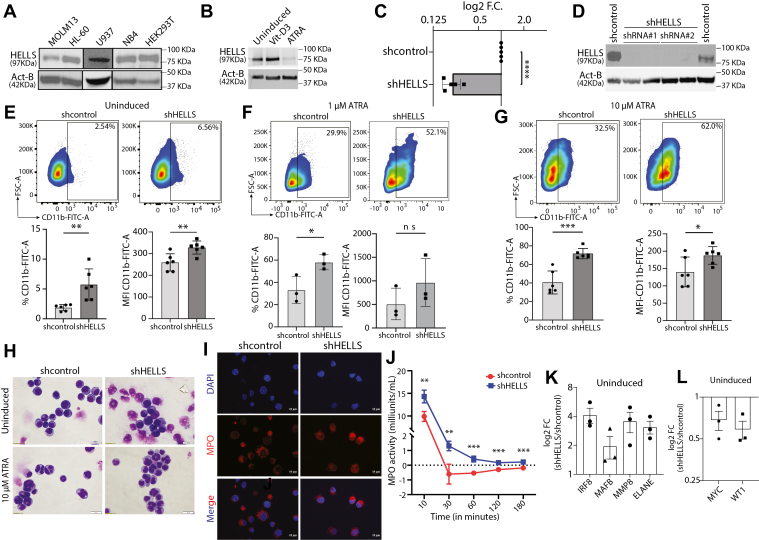
**Loss of *HELLS* promotes myeloid differentiation.***A*, total protein cell lysates (100 μg) from leukemic cell lines MOLM13, HL-60, U937, NB4, and HEK293T were used for immunoblotting for HELLS, and ACTB as loading control. Representative immunoblot images for leukemic cell lines and the HEK293T cell line. *B*, HL-60 cells were treated with 30 nM vitamin-D3 (Vit-D3) and 10 μM all-trans retinoic acid (ATRA) for 72 h. Total protein cell lysates (50 μg) from uninduced, Vit-D3, and ATRA were used for immunoblotting for HELLS and ACTB as a loading control. *C* and *D*, HL-60 cells were transduced with lentiviruses containing an empty vector (shcontrol) or shRNA targeting HELLS (shHELLS). HELLS knockdown was confirmed by qPCR analysis. ACTB was used as a housekeeping control (*C*). Total protein cell lysates (60 μg) from WT, shcontrol, and shHELLS were used for immunoblotting with HELLS, with ACTB as a loading control. Representative immunoblot images for HL-60 (*D*). *E*–*L*, flow cytometry analysis of HL-60 cells with shcontrol or shHELLS (SD = 3.353 ± 1.006) showing myeloid differentiation marker CD11b in uninduced (*E*), 1 μM (*F*), and 10 μM (G) ATRA for 72 h. Representative *pseudocolor plotswith grids*, showing percent negative and positive populations for single-stained CD11b-FITC populations. Median fluorescent intensity (MFI) plots of shcontrol and shHELLS HL-60 cells. *H*, representative Giemsa-stained images (20 μm) of shcontrol and shHELLS HL-60 cells in uninduced and 10 μM ATRA induction for 72 h. *I*, fluorescence imaging (10 μm) of shcontrol and shHELLS HL-60 cells. The sections were immune-stained with MPO (*red*), and DAPI (*blue*) was used to counterstain the nuclei. *J*, colorimetric analysis of myeloperoxidase activity in shcontrol and shHELLS HL-60 cells. *K* and *L*, relative log2 fold change of several (K) differentiation-related genes *IRF8* (1.294 ± 0.474), *MAFB* (0.8915 ± 0.5147), *MMP8* (1.482 ± 0.8557), *ELANE* (0.8153 ± 0.4707)) (L) leukemic transcription factors, *MYC* (0.187 ± 0.108), *WT1* (0.1488 ± 0.0859) in shHELLS HL-60 cells compared with shcontrol HL-60 cells. All statistical parameters used in this figure are for n ≥ 3 independent experiments; error bars are mean ± S.E.M. ∗*p* < 0.05, ∗∗*p* < 0.01, ∗∗∗∗*p* < 0.0001, two-tailed Student's *t* test. HELLS, helicase lymphoid-specific; qPCR, quantitative polymerase chain reaction.

To investigate the role of *HELLS* in cellular proliferation, myeloid differentiation, and leukemogenesis in AML, we examined HELLS expression levels in various leukemic cell lines and selected two leukemic cell lines, promyeloblast HL-60 and pro-monocytic U937, both of which showed high HELLS expression, for our *in vitro* studies (Fig. 2*A*). These cell lines can be differentiated into granulocytic and monocytic lineages using *all-trans* retinoic acid (ATRA) and vitamin D3, respectively (Figs. 2*B* and S3*A*). Granulocytic differentiation of HL-60 and U937 using ATRA significantly reduced HELLS expression (Figs. 2*B* and S3*A*). To evaluate the role of HELLS in leukemia cell differentiation, we first established lentiviral knockdown of HELLS in HL-60 and U937 cells, confirming knockdown at both the transcript and protein levels (Figs. 2, *C* and *D*, and S3, *B* and *C*). We checked self-differentiation and ATRA-induced myeloid differentiation capacity of leukemic cells in the absence of HELLS. The vector control (shcontrol) and HELLS knockdown (shHELLS) cells were stained with CD11b-FITC and subjected to flow cytometry analysis, which showed 3.1- fold and 5.3-fold higher percentage of CD11b positive and 1.3-fold and 1.4-fold higher median fluorescence intensity (MFI) in HL-60 and U937 cells, respectively, compared with that in the shcontrol (Figs. 2*E*, and S2, *D* and *E*). Retinoic acid induces granulocytic differentiation, and since loss of HELLS primed the cells for myeloid differentiation, we treated HL-60 and U937 cells with 1 μM and 10 μM ATRA, respectively, for 72 h to assess the enhanced differentiation potential conferred by shHELLS. The cells were scored for CD11b positivity by flow cytometry. Concentration-dependent myeloid differentiation was observed in HL-60 cells induced with a 10 times lower (1 μM) concentration of ATRA for 72 h. Flow cytometry was used to score for CD11b-positive cells. The lower concentration of ATRA also significantly enhanced the percentage of CD11b-positive cells in shHELLS (1.7-fold) compared with that in the shcontrol in HL-60 cells (Fig. 2*F*). HELLS depletion, in addition to ATRA treatment, significantly enhanced CD11b positivity compared with the shcontrol in HL-60 (1.8-fold) and U937 (1.8-fold) cells (Figs. 2, *F* and *G*, and S3, *F* and *G*). The nuclear morphology of undifferentiated and differentiated myeloid cells and leukemic blasts is well documented and routinely monitored to assess myeloid differentiation status and blast percentage in patients with AML (29). Giemsa staining showed deformed nuclear morphology, suggesting that loss of HELLS induces myeloid differentiation in leukemia. The nuclear morphological changes in shHELLS were more pronounced with ATRA induction than in shcontrol HL-60 and U937 cells (Figs. 2*H* and S3, *H* and *I*). To further validate ATRA-induced granulocytic differentiation, we evaluated myeloperoxidase (MPO) activity, a key enzyme involved in immune response in functionally competent granulocytes. MPO expression in the shHELLS HL-60 cells was higher than that in the shcontrol, as observed using an immunofluorescence assay (Fig. 2*I*). A time-course experiment further supported higher expression and activity of MPO in shHELLS compared with that in the shcontrol (Fig. 2*J*). We also validated the expression of myeloid lineage genes in shHELLS cells relative to shcontrol cells and observed upregulation of *IRF8*, *MAFB*, *MMP8*, and *ELANE* in HL-60 cells, whereas U937 cells showed upregulation of *IRF8* and *ELANE* (Figs. 2*K* and S3*J*). *MYC* and *WTI* were downregulated in shHELLS in HL-60 cells, and *MYC* was downregulated in shHELLS- U937 cells (Figs. 2*L* and S3*K*). The differences in gene expression fold changes between HL-60 and U937 cells could be attributed to cell type.

### Leukemic cells are arrested in the G0-G1 stage of the cell cycle in the absence of *HELLS*

**Figure 3:**
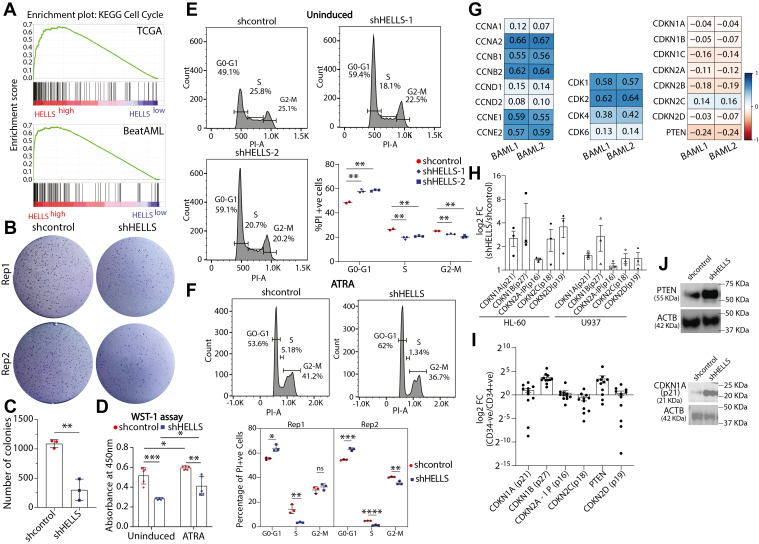
**Leukemic cells are arrested in the G0-G1 stage of the cell cycle in the absence of *HELLS*.***A*, AML patients from TCGA and the BeatAML cohort were stratified into the *top* (GSEA) and *bottom* 10th percentiles, as HELLS^High^ and HELLS^Low^ groups. Gene set enrichment analysis of transcriptomic signatures in HELLS^High^ and HELLS^Low^ groups from TCGA (*top panel*) and BeatAML (*bottom panel*) cohort. *B*, shcontrol and shHELLS knockdown HL-60 cells were seeded in 0.3% soft-agar. After 14 days of incubation, colony morphology and number were assessed by crystal violet staining and imaging using light microscopy. Two biological replicates are represented. *C*, *bar plot* showing the colony numbers of shcontrol (*red*) and shHELLS (*blue*) HL-60 cells from three biological replicates. *D*, shcontrol (*red*) and shHELLS (*blue*) HL-60 cells were seeded for cell proliferation assay using WST-1 assay. Bar plot showing the absorbance (450 nm) value from three technical replicates obtained from two biological replicates. *E*, flow cytometry analysis of PI-stained control or shHELLS showing cell cycle profile in uninduced cells. Representative *histogram plots* and *bar plots* showing percent positive populations for propidium iodide positive populations in different stages of the cell cycle. *F*, flow cytometry analysis of PI-stained control (*red*) or shHELLS (*blue*) showing cell cycle profile of HL-60 cells treated with 1 μM ATRA for 48 h. Representative *histograms* and *bar plots* show the percent positive populations obtained from propidium iodide-stained cells at different stages of the cell cycle. The data presented is from three replicates obtained from two independent HELLS knockdown experiments. *G*, heatmap depicting the correlation values of genes encoding cyclins, cyclin-dependent kinases, and cyclin-dependent kinase inhibitors with respect to *HELLS*, curated from BeatAML datasets. *H*, relative log2 fold change (three independent knockdown experiments) of several cyclin-dependent kinase inhibitors (*CDKN1A* (HL-60–0.9802 ± 0.5659, U937–0.1579 ± 0.09115), *CDKN1B* (HL-60–4.243 ± 2.450, U937–1.719 ± 0.9924), *CDKN2A-IP* (HL-60–0.8199 ± 0.0473, U937–0.1293 ± 0.0746), *CDKN2C* (HL-60–1.357 ± 0.7832, U937–0.3481 ± 0.2010), and *CDKN2D* (HL-60–1.731 ± 0.9997, U937–0.4412 ± 0.2547)) genes in shHELLS HL-60 and U937 cells compared with shcontrol HL-60 and U937 cells, respectively. *I*, relative log2 fold change of *PTEN* (1.425 ± 0.4297) and cyclin-dependent kinase inhibitors (*CDKN1A* (1.949 ± 0.5877), *CDKN1B* (13.06 ± 3.936), *CDKN2A-IP* (1.598 ± 0.4819), *CDKN2C* (0.7038 ± 0.2122), and *CDKN2D* (18.09 ± 5.455)) in CD34+ AML cells (n = 10) compared with that in the CD34− population (n = 11). *J*, total protein cell lysates (50 μg) from uninduced shcontrol and shHELLS HL-60 cells were used for immunoblotting for PTEN, p21 (CDKN1A), and ACTB as loading control. Representative immunoblot images for HL-60 shcontrol and shHELLS cells. All statistical parameters used in this figure are from biological and technical replicates as mentioned in each panel; error bars are mean ± S.E.M. ns - non-significant, ∗*p* < 0.05, ∗∗*p* < 0.01, ∗∗∗*p* < 0.001, two-tailed Student's *t* test. HELLS, helicase lymphoid-specific; AML, acute myeloid leukemia; TCGA: The Cancer Genome Atlas; ATRA, all-trans retinoic acid; CDKN, cyclin-dependent kinase inhibitor.

Loss of HELLS severely affects cellular proliferation in hepatocellular carcinoma and cervical and pancreatic cancers (4, 14, 33, 34). Additionally, differentiating HL-60 cells treated with ATRA shows reduced growth potential (29). As HELLS is essential for cellular proliferation and development, we hypothesized that its loss might affect the cell cycle, either directly or indirectly. We classified genes associated with high or low HELLS expression in TCGA and BeatAML datasets and performed Gene Set Enrichment Analysis (GSEA). The genes associated with HELLS^high^ showed significant enrichment for cell cycle genes compared with HELLS^low^ cells (Fig. 3*A*). We performed a soft agar assay, in which shHELLS cells showed significantly fewer colonies and smaller colony size than shcontrol cells (Fig. 3, *B* and *C*). Next, we quantified proliferation of shcontrol and shHELLS cells treated with ATRA for 48 h using the WST-1 assay. We found that shHELLS cells were significantly less proliferative than shcontrol cells both with and without ATRA (Fig. 3*D*). Further, we investigated cell cycle profiles in shHELLS and shcontrol cells using flow cytometry and found that the cells were arrested in G0-G1 and that 59.4% (*p*-value 1.0e-06) and 59.1% (*p*-value <1.0e-06) cells were not able to enter the S-phase or the G2-M phase (Fig. 3*E*). To further understand the effect of ATRA on the cellular proliferation of shcontrol and shHELLS cells, we investigated the cell cycle profile using flow cytometry and detected G0-G1 arrest (*p*-value = 1.34e-02) with significant reduction of S-phase (*p*-value = 1.41e-04) in shHELLS cells, indicating complete inhibition of the cell cycle (Fig. 3*F*). Next, we investigated the correlation of *HELLS* expression with representative regulators of the cell cycle, namely, cyclins (CCNs), cyclin-dependent kinases (CDKs), and cyclin-dependent kinase inhibitors (CDKNs) in AML patients using BeatAML1.0 and BeatAML2.0 curated from the cBioportal database. Since HELLS is associated with active cellular proliferation, we expected to observe a positive correlation with CCNs and CDKs and a negative correlation with CDKNs in the AML patient data. Indeed, *HELLS* showed a positive correlation with CCNs and CDKs and a negative correlation with CDKNs (Fig. 3*G*). Further, we attempted to validate a subset of CDKNs using reverse transcription-quantitative polymerase chain reaction (RT-qPCR) in *in vitro* and *ex vivo* models. The expression of *CDKN1A* (p21), *CDKN1B* (p27), *CDKN2A-IP* (p16), *CDKN2C* (p18), and *CDKN2D* (p19) was upregulated in both HL-60 and U937 in the absence of *HELLS* (Fig. 3*H*). In the *ex vivo* model, we isolated AML patient-derived CD34^+^ LSPCs (n = 9) and CD34^-^ (n = 10) cells and found similar upregulation of phosphatases and tensin homolog (*PTEN*) and *CDKN1A*, *CDKN1B*, *CDKN2A-IP*, and *CDKN2D* in the CD34^-^ population (Fig. 3*I*). However, we found downregulation of *CDKN2C*, a cyclin-dependent kinase inhibitor in the *ex vivo* model. Finally, we validated the upregulation of PTEN, a tumor suppressor gene, and CDKN1A, a cyclin-dependent kinase inhibitor essential for cell cycle arrest, at the protein level in HELLS-deficient cells (Fig. 3*J*). These results suggest that HELLS promotes leukemic cell proliferation by inhibiting the expression of cell-cycle regulators.

### Downregulation of *HELLS* promotes apoptosis in myeloid cells

**Figure 4:**
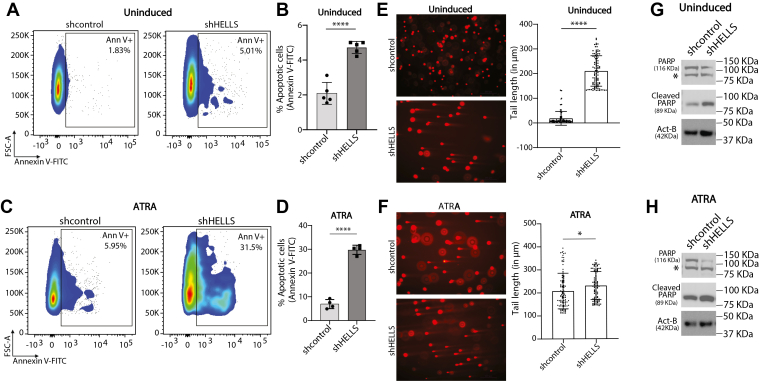
**Downregulation of *HELLS* promotes apoptosis in myeloid cells.***A* and *D*, flow cytometry analysis of HL-60 cells with shcontrol or shHELLS showing apoptotic marker Annexin V in uninduced (*A*) and 10 μM ATRA (*C*) for 72 h. Representative pseudocolor plots with grids of FACS data, showing percent positive populations for Annexin V positive populations (*A* and *C*). Bar plots showing percentage of Annexin V positive cells in shcontrol and shHELLS HL-60 cells (*B* and *D*). Representative alkaline comet assay image (200 μm) showing comet tail length in shcontrol and shHELLS HL-60 cells in (*E*) uninduced or 10 μM ATRA (F) for 48 h. The quantification of tail length for shcontrol (n = 100) and shHELLS (n = 100) is shown on the *right* side of the respective images. HL-60 shcontrol and shHELLS cells were treated with or without 1 μM all-trans retinoic acid (ATRA) for 48 h. Total protein cell lysates (50 μg) from uninduced (*G*) and ATRA-induced (*H*) shcontrol and shHELLS HL-60 cells were used for immunoblotting for PARP (∗ is cleaved PARP), cleaved-PARP (antibody specific for cleaved PARP), and ACTB as a loading control. Representative immunoblot images for HL-60. All statistical parameters used in this figure are for n ≥ 3 independent experiments; error bars are mean ± S.E.M. ns-not significant, ∗*p* < 0.05, ∗∗∗∗*p* < 0.0001 two-tailed Student's *t* test. HELLS, helicase lymphoid-specific; ATRA, all-trans retinoic acid; PARP, poly (ADP-ribose) polymerase.

Loss of HELLS sensitizes cells to cisplatin-induced apoptosis in several cancers, including pancreatic adenocarcinoma (33). Therefore, we used an Annexin V-based flow cytometry assay to identify the percentage of apoptotic cells in shHELLS cells. We found that loss of HELLS resulted in a 2.2-fold (shcontrol-2.092% ± 0.627 and shHELLS-4.726% ± 0.346) increase in the percentage of Annexin V-positive HL-60 cells (Fig. 4, *A* and *B*). Retinoic acid induction further enhanced the percentage of apoptotic cells in shHELLS cells (29.775% ± 1.936) compared with that in the shcontrol (6.935% ± 1.91), indicating that after HELLS knockdown, cells are predisposed to apoptosis (4.3-fold) (Fig. 4, *C* and *D*). A previous study reported that the loss of HELLS induces DNA damage in glioblastoma stem cells and that this DNA damage also predisposes the cells toward apoptosis (17). We were thus intrigued to investigate DNA damage levels in leukemic cells and performed single-cell gel electrophoresis, also known as the alkaline comet assay. Results revealed increased comet tail length in shHELLS cells (210.6 μm) compared to that in shcontrol (18.8 μm) HL-60 cells, suggesting that loss of HELLS resulted in higher DNA damage (Fig. 4*E*). Comet tail length increased by 1.11-fold in ATRA-treated shHELLS cells compared to that in shcontrol HL-60 cells. However, as ATRA also induces terminal granulocytic differentiation and apoptosis, the difference in the tail length between shHELLS and shcontrol is minimal (Fig. 4*F*). Therefore, the results of the alkaline comet assay suggest that loss of HELLS increases DNA damage, as evidenced by long comet tails in shHELLS cells. To further validate induction of apoptosis in the absence of HELLS, we used shHELLS and shcontrol in uninduced and ATRA-induced conditions to assess cleaved poly (ADP-ribose) polymerase (PARP) levels, a marker of apoptosis. The results of the immunoblotting assay indicate that cleaved PARP expression in shHELLS cells was higher than in shcontrol cells under both uninduced and ATRA-induced conditions, suggesting that HELLS deficiency induces apoptosis (Fig. 4, *G* and *H*).

### Loss of *HELLS* alters global histone modifications

**Figure 5:**
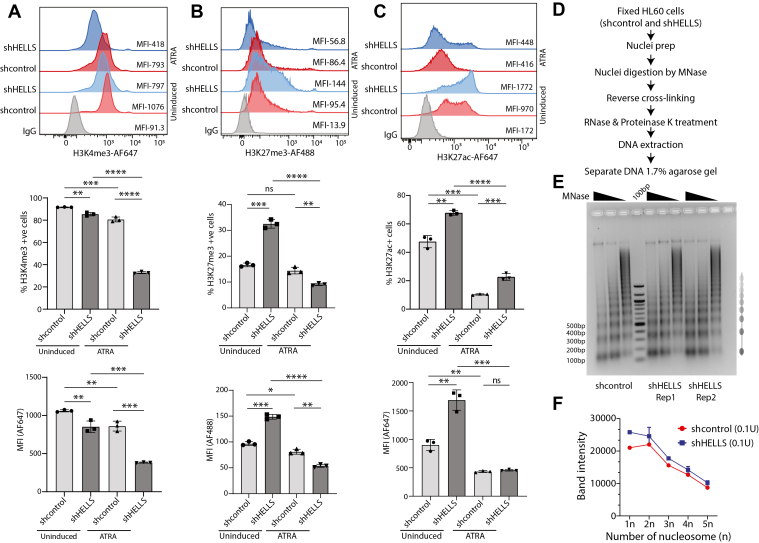
**Loss of *HELLS* alters global histone modifications.***A*, flow cytometry analysis of HL-60 cells with shcontrol or shHELLS showing active histone marks H3K4me3 on promoters in uninduced and 1 μM ATRA for 48 h. Median fluorescent intensity (MFI) plots of shcontrol and shHELLS HL-60 cells. *B*, flow cytometry analysis of HL-60 cells with shcontrol or shHELLS showing repressive histone mark H3K27me3 on promoters in uninduced and 1 μM ATRA for 48 h. Median fluorescent intensity (MFI) plots of shcontrol and shHELLS HL-60 cells. *C*, flow cytometry analysis of HL-60 cells with shcontrol or shHELLS showing active enhancer histone mark H3K27ac on enhancers in uninduced and 1 μM ATRA for 48 h. Median fluorescent intensity (MFI) plots of shcontrol and shHELLS HL-60 cells. *D*, schematic representation of the micrococcal nuclease (MNase) experiment. *E*, concentration-dependent (MNase) digestion in uninduced shcontrol and shHELLS HL-60 cells. Representative agarose gel image showing nucleosomal DNA ladder of mono-, di-, tri-, etc., nucleosomes in shcontrol and shHELLS HL-60 cells. *F*, *line graph* showing the quantification of band intensity for MNAse-digested ladder-like bands in shcontrol and shHELLS at the highest MNase concentration. The band intensity was analyzed using GelAnalyzer software. All statistical parameters used in this figure for n ≥ 3 independent experiments; error bars are mean ± S.E.M. ns-not significant, ∗*p* < 0.0.05, ∗∗*p* < 0.01, ∗∗∗*p* < 0.001, ∗∗∗∗*p* < 0.0001, two-tailed Student's *t* test. HELLS, helicase lymphoid-specific; ATRA, all-trans retinoic acid.

Epigenetic mechanisms modulate the gene regulatory landscape; therefore, we investigated global alterations in histone modifications in the absence of the HELLS chromatin remodeler. Reports indicate that posttranslational histone marks are altered in leukemia (35). We therefore investigated whether loss of *HELLS* in HL-60 cells altered histone modifications, with and without ATRA induction. We conducted flow cytometry analysis of active (H3K4me3) and repressive (H3K27me3) promoter marks, along with H3K27ac, a marker of active enhancers. The percentage and MFI of H3K4me3 were significantly reduced in shHELLS (85.43%, MFI = 853; *p*-value = 2.3e-03) cells compared to those in the shcontrol (91.67%, MFI = 1061) HL-60 cells. Similarly, ATRA-induction in shHELLS cells further reduced (32.87%, MFI = 383.3; *p*-value = 2.0e-04) H3K4me3 levels compared with those in the ATRA-induced shcontrol (80.67%, MFI = 860.7) cells (Figs. 5*A* and S4*A*). The levels of the repressive histone mark H3K27me3 were significantly elevated in shHELLS cells compared with shcontrol cells, suggesting a global shutdown of gene expression. However, ATRA-induced granulocytic differentiation did not increase the repressive promoter mark in shHELLS cells compared with shcontrol HL-60 cells (Figs. 5*B* and S4*B*). During cellular differentiation, primed enhancers become active, inducing lineage-specific transcription factors, and thereby acquire the H3K27Ac histone mark (35). Furthermore, during differentiation, local H3K27me3 levels decrease *via* active or passive mechanisms, whereas the enhancer mark H3K27Ac becomes enriched (36). Thus, we also measured H3K27Ac levels in shHELLS HL-60 cells. The shHELLS cells showed higher levels of H3K27Ac than the shcontrol HL-60 cells. However, ATRA treatment did not increase H3K27Ac levels in shHELLS HL-60 cells compared with shcontrol cells (Figs. 5*C* and S4*C*). The differences in the H3K27me3 repressive promoter mark and H3K27 acetylation mark could be attributed to granulocytic differentiation and warrant further investigation.

Next, we performed chromatin accessibility using micrococcal nuclease (MNase) to understand global chromatin dynamics upon losing *HELLS* (Fig. 5*D*). The abundance of mono and di-nucleosome levels in the shHELLS samples showed higher chromatin accessibility (Fig. 5*E*). The mononucleosome band intensity (0.1U Mnase) was quantified, which showed an increase in chromatin accessibility in shHELLS cells, suggesting that loss of HELLS renders chromatin more vulnerable to MNase digestion (Fig. 5*F*).

### Convergence of *HELLS* expression with clinical phenotypes

**Figure 6:**
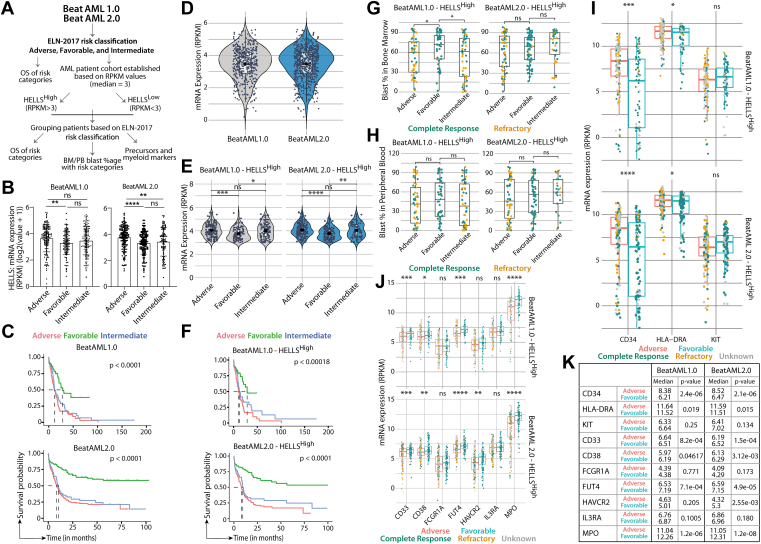
**High *HELLS* expression is a potential indicator of poor prognosis.***A*, workflow depicting the method followed in this study to understand the implication of upregulation of *HELLS* in AML patients by utilizing transcriptomic data available in cbioportal. *B*, bar plot showing *HELLS* mRNA expression in context of ELN-2017 risk classification in BeatAML1.0 and BeatAML2.0. *C*, overall survival of patients based on ELN-2017 classification with median survival time for adverse, favorable, and intermediate risk categories. *D*, violin plot representing AML patients cohort with high and low *HELLS* based on rounded median value of 3.58 and 3.59 for BeatAML1.0 and BeatAML2.0, respectively. A median of >3 and < 3 is used as a high and low *HELLS* gene expression. *E*, violin plot showing variation of HELLS^High^ cohort expression in adverse, favorable, and intermediate risk groups in BeatAML1.0 and BeatAML2.0 datasets. *F*, overall survival curves for ELN-2017 risk classification for HELLS^High^ cohort with corresponding median survival. *G* and *H*, box plot showing association of bone marrow and peripheral blood blast percentage in HELLS^High^ ELN 2017 risk groups. *I* and *J*, box plot representation of HELLS^High^ cohort with hematopoietic precursors, myeloid, and mature myeloid markers in adverse, and favorable risk categories. *K*, table summarizing the median and *p*-values for each gene comparison between adverse and favorable prognosis ELN classification. HELLS, helicase lymphoid-specific; AML, acute myeloid leukemia; ELN, European LeukemiaNet.

To further investigate the role of HELLS upregulation in AML patient samples and its relationship with leukemia pathology, we retrieved gene expression and metadata from cBioPortal. We curated a sample list of individual AML patients with *HELLS* expression and associated them with the European LeukemiaNet (ELN)-2017 and French-American-British (FAB) classifications, which categorize AML patients by prognosis and the maturation stage of AML blast cells, respectively. *HELLS* expression correlated with ELN-2017 classifications with respect to leukemic blast percentage, OS outcomes, and various hematological markers associated with HSCs and myeloid marker lineages (Fig. 6*A*). For French-American-British classification, we classified *HELLS* expression for each sub-types (M0, M1, M2, M3, M4, and M5) and the OS of patients with AML in each category (Fig. S5). We curated data on all AML patients with *HELLS* gene expression (RPKM values ranging from 0.2 to 5.6) in BeatAML1.0 and BeatAML2.0. In total, 672 samples (n = 451 with HELLS expression) in BeatAML1 and 942 samples (n = 671 with *HELLS* expression) in BeatAML2, with their follow-up duration, and the 2017 ELN classification data were curated for downstream study. The *HELLS* expression (RPKM) values in BeatAML1 and BeatAML2 were categorized according to the ELN-2017 risk classification in an unbiased manner. Interestingly, we found that the favorable risk group has significantly lower *HELLS* transcript levels. At the same time, the highest expression was observed in the adverse risk groups (with median values, for BeatAML1.0 Adverse = 3.81, Favorable = 3.30, Intermediate = 3.50, for BeatAML2.0 Adverse = 3.86, Favorable = 3.40, Intermediate = 3.51) (Fig. 6*B*). This observation suggests a potential correlation between *HELLS* expression and overall risk stratification in patients with AML. Therefore, we evaluated OS outcomes and HRs. The favorable risk group demonstrated the longest median survival time (28.6 months in BeatAML1, while the median survival of the favorable group was not available in BeatAML2, as >50% of the patients suffered from the event of death. In contrast, the adverse group showed the shortest survival (12.2 months in BeatAML1 and 8.4 months in BeatAML2) (Fig. 6*C*). The favorable group showed the lowest HRs, 0.37 and 0.31 in BeatAML1 and BeatAML2, respectively, compared with the adverse group (HR = 1), which acted as the reference group in both datasets (Table S1). This integrative approach may help determine differential gene expression of *HELLS* in relation to prognostic patterns, patient stratification, and leukemic phenotypes, thereby helping to understand survival outcomes in patients with AML.

We observed a wide range of *HELLS* gene expression (0.2–5.6 RPKM) in the BeatAML datasets, which made it difficult to correlate with AML pathology. Therefore, we stratified AML patients into two groups: one with high *HELLS* expression (HELLS^High^) and the other with low *HELLS*expression (HELLS^Low^). In this study, *HELLS* gene expression groups were defined using a rounded median RPKM cut-off of 3 (HELLS^High^; [median ≥3] and HELLS^Low^ [median ≤2.8]). The *HELLS* gene expression distribution for BeatAML1.0 (HELLS^High^ [n = 328] and HELLS^Low^ [n = 123]) and BeatAML2.0 (HELLS^High^ [n = 476] and HELLS^Low^ [n = 195]) AML patient cohorts were defined for downstream analysis (Fig. 6, *A* and *D*). Next, we evaluated risk distribution across ELN-2017-classified samples in the HELLS^High^ and HELLS^Low^ cohorts of BeatAML. The HELLS^High^ AML patient cohort in the adverse group showed significantly higher expression of *HELLS* (M = 3.99, *p*-value = 2.0e-04 in BeatAML1 and M = 4.01, *p*-value = 1.0e-05 in BeatAML2), while those in the favorable group displayed comparatively lower RPKM expression levels in both the BeatAML1 and BeatAML2 datasets (M = 3.63, *p*-value = 2.0e-04 in BeatAML1 and M = 3.75, *p*-value = 1.0e-05 in BeatAML2) (Fig. 6*E*). Interestingly, the median values of the adverse, favorable, and intermediate groups of HELLS^Low^ AML patients showed negligible differences, suggesting that low *HELLS* expression could not be used to distinguish between ELN classifications (Fig. S6 and Table S1). This supports the association of the HELLS^High^ cohort with adverse prognosis in AML. Finally, within the HELLS^High^ cohort, we evaluated survival outcomes and HR across the ELN risk categories. This analysis again revealed a similar trend where the favorable group retained the longest median survival (M = 28.6 months; *p*-value = 1.8e-05 in BeatAML1.0 and less than 50% death cases in BeatAML2.0), while the adverse group continued to show poor survival outcomes (M = 10.5 months in BeatAML1.0 and M = 8.05 months in BeatAML2.0) (Fig. 6*F*). These findings collectively support the association of high *HELLS* expression with adverse prognosis and its potential utility as a prognostic biomarker in AML.

### High *HELLS* expression is a potential indicator of poor prognosis

We further investigated the association between ELN risk categories within the HELLS^High^ cohort. We examined the percentages of bone marrow and peripheral blood (PB) blasts associated with disease severity by integrating clinical responses, such as complete response (CR) and refractory response (RR) (Fig. 6, *G* and *H*). Median blast percentages were insufficient to draw definitive conclusions regarding their relationship to ELN risk categories, possibly due to differences in treatment regimens. However, the favorable group (n = 61 in BeatAML1.0 and n = 83 in BeatAML2.0) in the HELLS^High^ cohort included patients who achieved CR, whereas the adverse-risk group (n = 38 in BeatAML1.0 and n = 43 in BeatAML2.0) did not. Conversely, most patients in the unfavorable group (n = 46 in BeatAML1.0 and n = 47 in BeatAML2.0) exhibited refractory responses (Fig. 6, *G* and *H*). These results suggest that, even among patients with high *HELLS* expression, ELN classification remains clinically relevant for AML stratification and predicts outcome.

To further investigate the clinical landscape of the HELLS^High^ cohort, we analyzed a subset of representative markers from hematopoietic precursors and the myeloid lineage as stratified by ELN risk groups (37). We found significantly high expression of *CD34* and *HLA-DRA* in the adverse ELN risk group, suggesting a possible enrichment of undifferentiated blast populations in these patients, which is associated with poor prognosis, poorer clinical outcomes, and higher blast burden (Fig. 6, *I* and *K*). In contrast, myeloid lineage markers, *CD33*, *CD38*, *FCGR1A*, *FUT4* (*CD15*), *HAVCR2*, *IL3RA*, and *MPO*, were highly expressed in the favorable ELN risk group, indicating a more differentiated myeloid phenotype (Fig. 6*J*). The overall assessment of all marker genes (medians and *p*-values) was performed in both BeatAML1.0 and BeatAML2.0 cohorts, yielding consistent results (Fig. 6*K*). High expression of myeloid lineage markers is commonly associated with better treatment responses and reduced disease progression in patients with favorable risk. It is interesting to note that in the HELLS^High^ cohort, the adverse group is positively associated with high expression of HSPC/LSPC markers and negatively associated with myeloid differentiation markers. In contrast, low *HELLS* expression in the favorable group is positively associated with high myeloid marker expression. Together, these datasets are indicative of *HELLS* upregulation as a potential prognostic marker for AML patients.

### Loss of *HELLS* in patient-derived CD34^+^ LSPCs reduces leukemic burden and increases apoptosis

**Figure 7:**
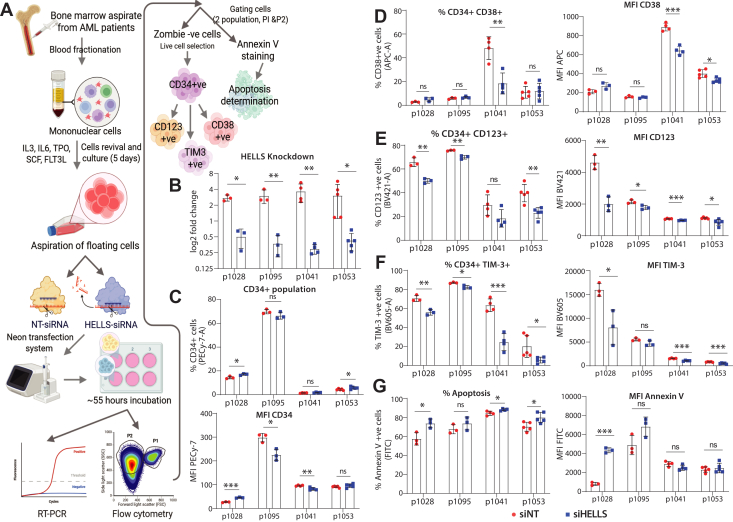
**Loss of *HELLS* results in diminished leukemic signatures and increased apoptosis in AML patient-derived LSPCs.***A*, schematic representation of the complete workflow of the *ex vivo* model, knockdown strategy, and flow cytometry analysis pipeline of the AML-patient-derived LSPCs experiments. Illustrations created using BioRender.com. *B*, AML patient-derived LSPCs were transfected with either non-target siRNA (siNT) or siRNA targeting HELLS (siHELLS). *HELLS* knockdown was confirmed by qPCR analysis for all patient-derived LSPCs (p1028, n = 3; p1095, n = 3; p1041, n = 4; and p1053, n = 5), where “n” represents the number of independent siRNA transfections for each patient sample that showed significant levels of *HELLS* knockdown. *ACTB* was used as a housekeeping control. *C*–*F*, bar plots showing percentage and MFI of CD34+, CD34+CD38+, CD34+CD123+, and CD34+ TIM-3+ cells in siNT (*red*) and siHELLS (*blue*) cells. *G*, bar plots obtained from flow cytometry analysis of AML-patient derived siNT (*red*) and siHELLS (*blue*) cells showing percentage positive and MFI of apoptotic marker Annexin V. All statistical parameters used in this figure are for n ≥ 3 independent siNT and siHELLS experiments; error bars are mean ± S.E.M. ns-not significant, ∗*p* < 0.0.05, ∗∗*p* < 0.01, ∗∗∗*p* < 0.001, ∗∗∗∗*p* < 0.0001 two-tailed Student's *t* test. HELLS, helicase lymphoid-specific; AML, acute myeloid leukemia; LSPC, leukemic stem/progenitor cell; MFI, median fluorescence intensity.

Using big data, we showed that *HELLS* upregulation in AML patient-derived LSPCs correlates with higher expression of CD34 stem/progenitor markers and lower expression of CD38 myeloid markers. To further validate the effect of *HELLS* in AML patient-derived LSPCs, we performed siRNA-mediated HELLS knockdown and measured levels of various leukemic markers in the CD34^+^ population by flow cytometry. Briefly, AML patient-derived mononuclear cells (MNCs) were cultured, and suspension cells were transfected with siRNA using the Neon transfection system. The transfected cells were cultured for ∼55 h, evaluated for *HELLS* knockdown levels using RT-PCR, and then analyzed by flow cytometry (Fig. 7*A*). Compared with the nontarget siRNA (siNT), the HELLS-specific siRNA (siHELLS) showed significant knockdown in patient-derived LSPCs (Fig. 7*B*). These samples were assessed for CD34, CD38, CD123, TIM3, Annexin V, and Zombie dye (for dead cells). The flow cytometry analysis strategy is explained in Fig. S7. Forward and side scatter revealed two distinct cell populations (P1 and P2), which were analyzed in detail to determine the total number of events and the percentage of the cells present in P1 and P2 (Figs. S7 and S8, *A* and *B*). The P1 population is a minor population for siNT (3–9%) and siHELLS (7–15%), compared with the P2 population (siNT = 46–69% and siHELLS = 46–65%) (Figs. S7 and S8). Therefore, we focused on the major cell population (P2) to measure live cells and assess other cell-surface markers. The CD34^+^ population was identified, and the percentages and MFI values of CD38, CD123, and TIM3 were measured (Fig. 7 and S7). We observed that the percentage and MFI of CD34^+^ cells in siHELLS were significantly affected in some but not all the samples (Fig. 7*C*). Moreover, the CD38^+^ cells did not show enrichment upon HELLS knockdown; in fact, CD38 levels were reduced in two patient samples (Fig. 7*D*). Next, we checked CD123 and TIM3 expression, which are well-known LSC markers in AML (38, 39). They are both overexpressed in AML but not in normal HSCs (39, 40). Patient-derived CD34^+^ cells, together with CD123-and TIM3-positivity, constitute a LSC signature. HELLS knockdown resulted in a significant reduction in CD123-and TIM3-positive cells, suggesting a decrease in the leukemic burden in AML patient-derived LSPCs (Fig. 7, *E* and *F*). Further, siHELLS cells showed a significant increase in Annexin V-positive cells, indicating enhanced apoptosis, similar to that observed *in vitro* in leukemic cell lines (Fig. 7*G*). These data suggest that HELLS is essential for the survival and proliferation of leukemic cells and that HELLS deficiency reduces leukemic burden.

### *HELLS* depletion enhances drug-induced myeloid differentiation and apoptosis

**Figure 8:**
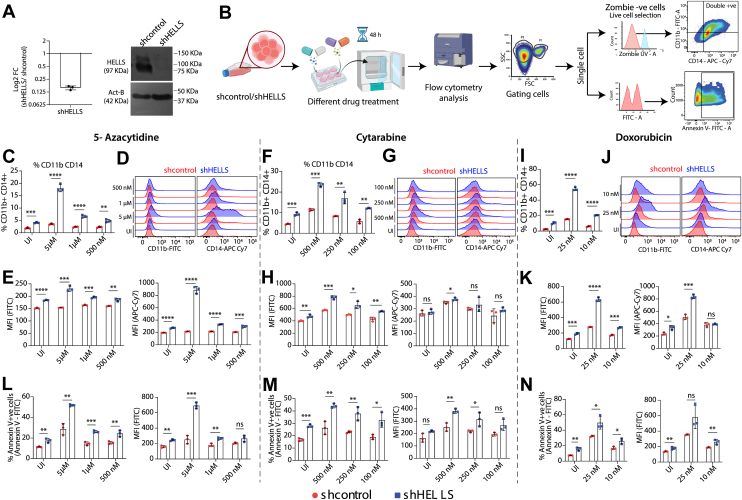
**HELLS depletion enhances drug sensitivity in leukemic cells.***A*, HL-60 cells were transduced with lentiviruses containing an empty vector (shcontrol) or shRNA targeting HELLS (shHELLS). *HELLS* knockdown was confirmed by qPCR analysis. *ACTB* was used as a housekeeping control. Representative immunoblot from shcontrol, and shHELLS confirming HELLS knockdown, with ACTB as a loading control. *B*, model showing the overall workflow of drug treatment strategy in shcontrol and shHELLS. The chemotherapeutic drugs, 5-azacytidine, cytarabine, or doxorubicin, were treated for 48 h. Illustrations created using BioRender.com. *C*–*K*, flow cytometry analysis showing cell percentages (double-positive cells for CD11b + -FITC and CD14+-APC-Cy7) and MFI for myeloid markers for shcontrol (*red*) and shHELLS (*blue*) with different concentrations of 5-azacytidine (5 μM, 1 μM, and 500 nM), cytarabine (500 nM, 250 nM, and 100 nM), and doxorubicin (25 nM and 10 nM). Representative histograms (*D*, *G*, and *J*) for each drug concentration from one induction replicate showing the median fluorescence intensity of CD11b-FITC-A and CD14-APC-Cy7-A for shcontrol (*red*) and shHELLS (*blue*). *L*–*N*, bar plots showing percentage of Annexin V positive cells in shcontrol (*red*) and shHELLS (*blue*) for different concentrations of 5-azacytidine, cytarabine, or doxorubicin drug treatment in HL-60 cells. All statistical parameters used in this figure are for n = 3 independent drug induction studies for shcontrol and shHELLS experiments; error bars are mean ± S.E.M. ns-not significant, ∗*p* < 0.0.05, ∗∗*p* < 0.01, ∗∗∗*p* < 0.001, ∗∗∗∗*p* < 0.0001 two-tailed Student's *t* test. HELLS, helicase lymphoid-specific; qPCR, quantitative polymerase chain reaction.

Since HELLS-deficient cells show enhanced myeloid differentiation and apoptosis in response to retinoic acid, we aimed to determine whether loss of HELLS sensitizes leukemic cells to other chemotherapeutic drugs. We first treated HL-60 cells with various concentrations of 5-azacytidine, cytarabine, and doxorubicin to determine IC_50_ values, which were found to be 12.2 μM, 2.2 μM, and 49 nM, respectively (Fig. S9, *A*–*C*). The experimental design and flow cytometry analysis workflow for the highest concentrations of each drug treatment in shcontrol and shHELLS are shown in Figures 8, *A* and *B* and S10. Next, we treated shcontrol and shHELLS with different concentrations (≥2-fold lower concentration with respect to IC_50_ values) of these drugs and evaluated their myeloid differentiation potential (double positive CD11b and CD14), and apoptosis (Annexin V positivity) (Fig. 8). The drug concentrations used in this study did not show a significant percentage of dead cells (Zombie-UV-positive cells). However, we observed that drug treatment significantly increased myeloid differentiation (CD11b+ and CD14+ cell percentages and MFI) in a concentration-dependent manner (Fig. 8, *C*–*E*, *F*–*H*, and *I*–*K*). In a similar manner, we observed significant increases in apoptotic cells with drug treatment (Fig. 8, *L*–*N*). Overall, these data suggest that loss of HELLS sensitizes leukemic cells to chemotherapeutic drug response. Further investigation is required to fully understand the underlying mechanisms for chemosensitization in HELLS-deficient cells and to develop an effective therapeutic regimen for patients with AML.

## Discussion

*HELLS*, is an essential factor for normal development and is highly expressed in proliferating tissues, while its loss results in lethality in mice (3). Upregulation of HELLS has been reported in several solid tumors, where it promotes proliferation and progression (4); however, its role in hematological cancers remains poorly understood. In this study, we show HELLS is a key epigenetic factor for normal myelopoiesis and is pathologically overexpressed in AML. We demonstrated that *HELLS* expression is abnormally elevated in AML-derived CD34+ LSPCs compared with normal myeloid differentiation, where its expression is reduced. HELLS deficiency promoted myeloid differentiation, cell cycle arrest, apoptosis, chromatin remodeling, and chemosensitivity. Big data analysis reveals that high *HELLS* expression is associated with poor prognosis and refractory AML, while its depletion reduces leukemic marker expression, including CD123 and TIM-3. Overall, our findings strongly suggest that HELLS contributes to the maintenance of an undifferentiated leukemic state and is linked with adverse prognosis, making it a potential prognostic marker and therapeutic target in AML.

SNF2 ATPases, which primarily mediate chromatin remodeling, exhibit altered phenotypes in cancers, thereby disrupting the epigenetic landscape. The upregulation of HELLS and the incidence of increased proliferation have been reported across several solid tumors (21, 34, 41). Interestingly, *HELLS* is also upregulated in AML with a possible role in cancer progression. In agreement with these studies, GSEA and correlation analyses of AML patients’ transcriptomic data revealed a positive correlation of cell cycle genes with upregulated *HELLS* expression. Further HELLS depletion in our model increased expression of cell cycle-inhibitory genes, such as p21 and PTEN, accompanied by impaired colony formation and cell cycle arrest. These observations support a role for HELLS in sustaining leukemic proliferative fitness. The LSPCs are maintained by promoting the expression of genes associated with cancer stemness and by repressing differentiation genes *via* multiple epigenetic mechanisms (26). *HELLS* is one of these genes whose upregulation alters epigenetic mechanisms that promote cell proliferation, block apoptosis, and inhibit myeloid differentiation (4). A similar phenotype is observed in retinoblastoma, lung adenocarcinoma, pancreatic cancer, and colorectal cancer, where loss of *HELLS* limits tumor progression and promotes the OS of cancer patients, demonstrating upregulation of *HELLS* as a key cancer phenotype and a prognostic marker (33, 42, 43, 44).

The proliferation-associated SNF2-like gene (PASG) or HELLS was identified as an essential factor upregulated in proliferating cells and downregulated in nonproliferating cells (25). Similarly, we found upregulation of the *HELLS* mRNA in HSPCs, and its expression decreased in differentiated terminally myeloid cells. The downregulation of *HELLS* during myeloid differentiation correlates with reduced chromatin accessibility, indicating that *HELLS* is not only essential for maintaining a normal HSPC pool but that epigenetic mechanisms also regulate its gene expression. An *in vitro* myeloid differentiation model further strengthens the role of HELLS in HSPCs, as its knockdown predisposes leukemic cells to myeloid differentiation. HELLS-depleted cells exhibited elevated expression of differentiation markers, including CD11b, MPO, *MMP8*, *ELANE*, *IRF8*, and *MAFB*, accompanied by distinct nuclear morphological changes characteristic of myeloid maturation. ATRA treatment further enhanced differentiation in those cells, suggesting that HELLS depletion makes the leukemic cells more responsive toward differentiation-inducing signals. Previous reports have demonstrated that HELLS acts as a key factor in cancer cell survival by evading DNA damage and apoptosis (4, 7, 45). In addition to regulating proliferation, HELLS appears to contribute to leukemic cell survival and genomic stability. HELLS-deficient cells exhibited increased apoptosis and elevated cleaved PARP levels, suggesting enhanced DNA damage and impaired survival signaling. In AML, the elevated persistence of *HELLS* expression may therefore sustain leukemic stemness and survival while suppressing lineage commitment.

Multiple direct and indirect mechanisms have been reported to regulate *HELLS* overexpression in various cancers. E2F transcription factors act as coactivators and cooperate with HELLS in tumorigenesis. Interestingly, loss of HELLS cannot be compensated by E2F3 overexpression, suggesting that HELLS may have independent functions (17). Pathways such as Sonic Hedgehog-YAP1 and the LRP6-GSK3β-E2F1 axis enhance *HELLS* promoter activity in medulloblastoma and glioblastoma, respectively (17, 46, 47). Tumor suppressors such as p53 indirectly regulate *HELLS* expression through the p21-FOXM1 axis, where the loss of p53 leads to FOXM1-mediated HELLS upregulation in hepatocellular carcinoma (15). HELLS can also repress the tumor suppressor TGFBR3 by binding its promoter in pancreatic cancer (33). Our results are consistent with elevated levels of P21 and PTEN in HELLS knockdown promyelocytes and in the CD34-ve population of patients with AML. Together, these findings suggest that *HELLS* upregulation can occur through tumor-specific networks and contribute to cancer progression.

The epigenetic landscape of histone modifications is altered during stem cell lineage commitment and maturation. Wong *et al.* demonstrated that the level of global hypermethylated H3K4 residues in LSCs is reduced upon differentiation due to lysine demethylation by KDM5B, thereby reducing their oncogenic potential (48). Interestingly, the H3K27me3 landscape in leukemia did not undergo significant genome-wide changes compared with that of the active promoter mark (35). Since *HELLS* mutations are rare in cancers, its oncogenic role is likely mediated through epigenetic dysregulation. HELLS-deficient promyelocytes showed a reduction in the H3K4me3 active mark and a concomitant increase in the H3K27me3 repressive mark. The promoter regions of stemness-related genes and lineage-determining genes exhibit opposing epigenetic landscapes, in which stemness-related genes lose active histone marks and acquire repressive histone marks, indicative of differentiation (35). Interestingly, we also observed greater enrichment of active enhancer histone marks and increased nuclease sensitivity in HELLS-deficient leukemic cells. However, since the simultaneous reduction in H3K27me3 and the enrichment of the active enhancer histone mark, H3K27Ac, are characteristic of a differentiated state, our results on changes in histone mark levels support the myeloid differentiation fate observed under conditions of HELLS deficiency (35). HELLS can also directly repress the tumor suppressor CDKN2A (p16), a cyclin-dependent kinase inhibitor, by recruiting histone deacetylases to its promoter, indicating a direct role in tumor initiation and progression (49). A previous report suggests that HELLS directly regulates nucleosome occupancy at the promoters and enhancers of multiple tumor suppressor genes in hepatocellular carcinoma (13). Furthermore, in differentiated embryonic stem cells, HELLS is required for the nucleosome occupancy at repeat sequences (50). It is noteworthy that cross-talk between multiple posttranslational histone modifications, along with their interactions with writers and erasers, modulates chromatin accessibility and defines cell fate and lineage commitment. Further investigation is required to understand how HELLS modulates epigenetic writers and erasers, directly or indirectly, in leukemogenesis.

Upregulation of HELLS is associated with the progression of several cancers and is linked with poor disease prognosis in multiple cancers (14). We observed that AML patients with low *HELLS* expression have a favorable prognosis and a high rate of complete remission. Furthermore, the combination of HELLS^High^ gene expression and adverse ELN risk constitutes an AML signature associated with higher disease burden and poorer treatment response. Additionally, the occurrence of a CR in the favorable risk group suggests that some patients with high *HELLS* expression may still benefit from standard therapy if their underlying cytogenetic or molecular risk is low. LSPCs exhibit higher expression of TIM-3 and CD123 than normal HSCs, which correlates with poor OS and failure to achieve CR after chemotherapy, thereby acting as a hallmark (38, 39, 40). We observed significant downregulation of CD123 and TIM-3 markers in AML patient-derived LSPCs following siRNA-mediated reduction in HELLS expression, indicating reduced leukemic burden. This highlights the potential of HELLS expression as a valuable biomarker, particularly when combined with established prognostic tools such as ELN, offering enhanced specificity for patient risk classification and response prediction.

It is noteworthy that although all patients with AML belonged to the HELLS^high^ expression group, the expression patterns of HSPCs and myeloid lineage markers aligned with ELN risk stratification. The CD marker’s association with ELN risk groups within a HELLS^high^ cohort supports the biological significance of HELLS as a clinically useful indicator of disease intensity. These findings support our model, in which HELLS upregulation contributes to a poor prognosis, although its impact is influenced by the disease’s underlying differentiation state and risk category. It also provides an additional layer of specificity for AML risk stratification and therapeutic targeting, ultimately helping identify targetable leukemic subpopulations within homogeneous high-risk molecular subgroups. This suggests that HELLS upregulation does not compromise the immunophenotypic and biological features associated with ELN risk groups, but rather may act synergistically with the established ELN-associated cellular phenotypes. Liang *et al.* have highlighted that overexpression of HELLS may promote a pro-tumorigenic and immunosuppressive Th2 response, thereby contributing to a poor prognosis (14). This indicates that upregulation of HELLS may promote tumorigenesis and poor prognosis in multiple ways, highlighting HELLS as a potential independent prognostic biomarker.

Hou *et al.* showed that loss of HELLS sensitizes pancreatic adenocarcinoma tumors toward cisplatin treatment and reduces overall tumor burden (33). Chemotherapeutic drugs such as 5-Azacytidine, Cytarabine, and Doxorubicin are routinely used for treating AML (51, 52). Another *in vitro* study shows that prolonged exposure to 5-azacytidine (Aza) induces differentiation by increasing MPO levels (51). Doxorubicin and Cytarabine, chemotherapeutic agents, are often used in the 7+3 regimen for AML (52). We found that treatment of these drugs in HELLS-deficient cells enhanced their sensitivity, promoting myeloid differentiation and apoptosis. In addition to these drugs, we also show that ATRA treatment not only reduces HELLS expression but also enhances apoptosis. Collectively, these findings suggest that targeting HELLS may improve therapeutic responsiveness.

Although our study has identified HELLS as a potential epigenetic regulator of leukemia, it lacks in-depth mechanistic molecular insights. The interdependence of HELLS with several cellular processes, such as proliferation and the cell cycle, warrants further investigation. Since HELLS is a key epigenetic factor that modulates normal cellular processes, the epigenetic mechanisms regulating its expression/overexpression need to be understood for therapeutic interventions. Despite these limitations, our study provides key insights into the role of HELLS in leukemic progression. To sum up, our study identifies HELLS as a key epigenetic regulator that supports leukemic stemness, proliferation, and survival while sabotaging myeloid differentiation in AML. In a clinical scenario, *HELLS* overexpression is linked with adverse clinical outcomes, refractoriness, and maintenance of LSPC-associated features, whereas its attenuation promotes differentiation, apoptosis, and enhanced drug sensitivity. Collectively, these findings position HELLS as a promising prognostic biomarker and potential therapeutic target in AML.

## Experimental procedures

### *In vitro* and *ex vivo* cell culture and myeloid differentiation

AML cell lines, HL-60 (CCL-240) and U937 (CRL-1593.2), were procured from the American Type Culture Collection and maintained under the culture conditions recommended by the American Type Culture Collection (www.atcc.org). MNCs were isolated from whole blood using Lymphoprep (Stem Cell Technologies) and from 30 to 50 ml of cord blood or 1 to 2 ml of AML patient-derived bone marrow cells, followed by isolation of CD34^+^ HSPCs and LSPCs using the CD34 microbead kit (Miltenyi Biotech, 130,046,703) per the manufacturer’s protocol. The isolated HSPCs were cultured in *ex vivo* media (Lonza, 04–743Q) supplemented with the recommended concentrations of cytokines such as IL3 (10 ng/ml), IL6 (10 ng/ml), SCF (100 ng/ml), TPO (20 ng/ml), and FLT3-L (100 ng/ml). The purity of the CD34^+^ HSPCs was monitored using flow cytometry (53). For all *ex vivo* knockdown experiments, total MNCs were isolated using Hi-Sep (Himedia, LS001), then centrifuged and frozen in a vital state. HL-60 and U937 cells were differentiated using the indicated amounts of ATRA for 72 h. Drug treatments were carried out using indicated concentrations of 5-Azacytidine (5 μM, 1 μM, 500 nM), Cytarabine (500 nM, 250 nM, 100 nM) and Doxorubicin (25 nM, 10 nM) for 48 h. Myeloid differentiation was assessed by flow cytometry of double-positive CD11b + cells and CD14+ cells. Cord blood-derived CD34+ HSPCs were differentiated using Myelocult (Stem Cell Technologies, 5150) supplemented with 1 μM hydrocortisone, along with 20 ng/ml M-CSF (Prospec, CYT-308) or 50 ng/ml GM-CSF (Prospec, CYT-221) for 9 to 13 days.

### Lentivirus preparation, transduction, and transfection

HEK293 T cells were transfected with a mixture of pMD2G, pCMVR, and pLKO empty plasmid or shRNAs targeting HELLS (TRCN0000273147, TRCN0000000306) or control (SHC001, Sigma-Aldrich) using the Calphos Mammalian Transfection kit (Takara, 631312) per the manufacturer’s instructions. The collected virus supernatant was used to transduce HL-60 or U937 cells. The transduced cells were selected using puromycin (1 μg/ml) for 96 h. For transfection using the *ex vivo* model, vital frozen cells were thawed at 37 °C, followed by the addition of prewarmed Iscove’s minimal essential medium (Gibco, 12440-046) drop-wise to a final volume of 10 ml. The cells were centrifuged at 300*g* for 15 min, and the pellet was washed with another 10 ml of media. Finally, the MNCs were suspended in 2 to 3 ml of *ex vivo* media and incubated for 48 to 72 h for revival, followed by transfection. siRNA transfections were performed using 50 nM nontarget control siRNA (Sigma-Aldrich, SIC001), siRNA targeting *HELLS* (Sigma-Aldrich, SASI_Hs01_00026586), 20 nM of silencer select negative control (Ambion, 4390843), or a mixture of silencer select siRNAs targeting *HELLS* (Ambion, s6505 and s6507). The MNC suspension (0.5 × 10^4^) was centrifuged and resuspended in T-buffer containing siRNA for transfection. Transfection was optimized at 1500 V, 10 ms, and 3 pulses using a Neon electroporation system (Thermo Fisher Scientific). Transfection with siNT (nontarget) or siHELLS (HELLS-specific) was performed four to six times, and three or more replicates were taken for downstream analysis.

### Flow cytometry assessment

The myeloid differentiation potential of HL-60 and U937 cells into granulocytes or monocytes was assessed by staining the cells with CD11b-FITC and CD14- APC-Cy7 antibodies in FACS buffer (1 mM EDTA, 2% fetal bovine serum in PBS. AML patient-derived LSPCs were assessed by staining the cells with CD34-PECy7, CD38-APC, CD123-BV421, and TIM3-BV605 antibodies in FACS buffer. The AML patient-derived LSPCs were stained with Zombie-UV dye to identify the live and dead cells. To determine the number of apoptotic cells in both cell lines and patient-derived cells, cells were stained with Annexin V-FITC in 1 × Annexin binding buffer (Invitrogen, V13246) and scored for FITC-positive cells. The data for differentiation assays and apoptosis were acquired at the Institute’s FACS central facility using a BD LSR Fortessa SORP cell analyzer (BD Biosciences) and analyzed using FlowJo software v10.7.2. A detailed list of antibodies used is provided in Table S3.

### RNA extraction and RT-qPCR

Total RNA was extracted using a DirectZol RNA isolation kit (Zymo Research, R2053) following the manufacturer’s instructions. The quality and concentration of RNA were measured using a spectrophotometer (MultiscanGO, Thermo Fisher Scientific). Total RNA was then converted to cDNA using the Maxima First Strand cDNA Synthesis Kit (Thermo Fisher Scientific, K1672) according to the manufacturer's instructions. RT-qPCR reactions were set using SYBR master mix (Applied Biosystems, A25778) in QuantStudio 6 Flex/3 (Applied Biosystems). *ACTB* was used as a housekeeping control, and relative expression levels were calculated using the 2^-ΔΔct^ method. The oligonucleotide sequences used are listed in Table S4.

### Morphological analysis of the nucleus using Giemsa staining

In total, 1.5 to 2 × 10^4^ cells were used for cytospin at 450 rpm for 5 min. The cell spot was air-dried and fixed with 100% methanol. May-Grunwald Giemsa stain was used to assess nuclear morphology. The cell morphology was evaluated using an Olympus IX83 microscope.

### MPO activity assay

MPO activity was measured using the MPO activity assay kit (MAK068, Sigma-Aldrich). Briefly, HL-60 shcontrol and shHELLS cells (2 × 10^6^) were homogenized in 4 volumes of MPO assay buffer. The cells were centrifuged, and the supernatant was used in triplicate to measure MPO activity at different time points. The MPO substrate was added to all samples, which were incubated at room temperature. At the desired time points, a stop mix was added to the samples to halt MPO activity, and the TNB reagent was then added to develop color. After further incubation, absorbance was measured at 412 nm (A_412_). The MPO activity was measured according to the manufacturer’s instructions.

### Colony-forming assay

In total, 2 × 10^3^ cells were mixed with culture media containing 0.3% low-melting agar (Sigma-Aldrich, A9414) and layered over culture media mixed with 0.6% low-melting agar in a 6-well plate. Cells were then cultured in a CO_2_ incubator at 95% humidity and 37 °C for 3 weeks, with fresh complete medium replenished every other day. The colonies formed were stained with 0.05% crystal violet solution. The images of the colonies and plates were captured using an Olympus IX83 microscope and a Chemidoc MP imaging system (Bio-Rad), respectively.

### WST-1 cell proliferation assay

Six replicates of HL-60 cells (10 × 10^4^ cells per well) in a 96-well plate were used for the proliferation assay. ATRA (1 μM) was added to each well and incubated at 48 h in an atmosphere of 5% CO_2_, 95% humidity, and 37 °C along with the control (no ATRA). After 48 h, 10 μl of WST-1 reagent (Sigma-Aldrich, 11644807001) was added to all wells, including the blank wells (only media), and the mixture was incubated under the same conditions for 3 h. Absorbance was measured at 450 nm using a MultiSkan GO (Thermo Fisher Scientific) spectrophotometer. The values were plotted after subtracting from the blank.

### Protein extraction and immunoblotting

HL-60 or U937 cells (5 × 10^6^) were lysed in RIPA buffer (150 mM NaCl, 50 mM Tris-HCl pH 8.0, 5 mM EDTA, 1% NP-40, 0.5% sodium deoxycholate, and 0.1% SDS) supplemented with protease inhibitors, 1 mM PMSF, 2 mM elastase inhibitor, and 1× Phosstop, followed by 10 cycles of sonication using Biorupter Pico (Diagenode). The Coomassie Plus Assay Kit (Pierce, 1856210) was used to estimate the protein concentration of the isolated protein, using bovine serum albumin (BSA) as the standard. Protein (30–50 μg) was separated on a 4 to 12% Bis-Tris polyacrylamide gel and transferred onto a polyvinylidene fluoride membrane (Immobilion, IPVH00010). Ponceau S staining was used to confirm protein transfer. The blot was then blocked using 5% BSA solution in Tris-buffered saline-Tween 20/1% casein solution and incubated overnight with the primary antibody at 4 °C, followed by washing and incubation with anti-rabbit or anti-mouse horse radish peroxidase-conjugated antibodies, as required. The blots were visualized in a Chemidoc (Biorad) using ECL solution or SuperSignal West Femto PLUS (Pierce, 34095). A detailed list of the antibodies used is provided in supporting information (Table S3).

### Immunofluorescence assay

Monolayer cells (15–20 × 10^3^) were used for cytospin and fixed using 3% formaldehyde solution (Pierce, 28,906) in PBS. Cells were washed and permeabilized with 0.01% Triton X-100 and then washed again with PBS. The cells were then blocked with 1% BSA and incubated overnight at 4 ° with the desired antibodies (1:250 dilution). The cells were then washed 3 times in PBS for 5 min each, followed by incubation with anti-rabbit-AF568 (Invitrogen, A-11036) and anti-mouse-AF488 (Invitrogen, A-21202) antibodies. DAPI antifade Gold (Invitrogen, P36935) was used as a mounting agent. The cells were imaged and analyzed using a Zeiss Apotome microscope.

### Alkaline comet assay

2.0 × 10^5^/ml PBS-washed cells were taken and resuspended again in PBS. Slides were precoated with 1% low-melting agarose (Sigma-Aldrich, A9414) and air-dried. The cell suspensions were mixed with low-melting agarose to a final concentration of 1%. The mixture was then spread on the precoated slide, covered with a coverslip, and immediately placed on ice to solidify. The coverslips were then removed gently by sliding. All procedures were henceforth performed under chilled conditions. The gel slides were then incubated in lysis buffer (2.5 M NaCl, 100 mM EDTA, 10 mM Tris-HCl, NaOH, pH 10) for 2 h, supplemented with 1% Triton X-100 and 1% dimethyl sulfoxide (DMSO). After lysis, the gel slides were incubated in the electrolysis buffer (1 mM EDTA, 1% DMSO, NaOH, pH>13) for 20 min and electrophoresed at 300 mA for 45 min in chilled electrophoresis buffer. The gel slides were then removed from the chilled electrophoresis buffer and incubated in the neutralization buffer (500 mM Tris-HCl, pH-7.5) for 20 min. The gel slides were then stained with 2.5 μM/ml propidium iodide in water and imaged using a Leica microscope. The images were analyzed for comet tail length using the CASP version 1.2.3b1 software (Sourceforge, Diceholdings Inc).

### Cell cycle analysis

In total, 1 × 10^6^ shcontrol and shHELLS HL-60 cells were taken after passage 5 to 8 and incubated with and without 1 μM ATRA for 48 h. The cells were then washed with PBS and fixed with 70% ethanol at 4 °C for 2 h. After fixation, the cells were washed twice with FACS buffer and stained with a mixture of Triton X-100, RNase, and propidium iodide for 45 min. The cells were then assessed for cell cycle stages using a BD LSR Fortessa (BD Biosciences) and analyzed with FlowJo software V 10.7.2.

### MNase assay

Five million cells each of the shcontrol and shHELLS HL-60 cells were fixed with 1% formaldehyde and used for MNase digestion. Cells were treated with MNase lysis buffer (10 mM Tris-HCl pH-7.5, 10 mM NaCl, 3 mM MgCl_2_, 0.5 mM spermidine, and 0.15 mM spermine) supplemented with 0.5% NP-40 and protease inhibitor (2×), followed by MNase digestion buffer (10 mM Tris-HCl pH-7.5, 15 mM NaCl, 60 mM KCl, 0.5 mM spermidine, and 0.15 mM spermine) supplemented with 0.5 mM PMSF and 1 M calcium chloride. MNase (N5386, Sigma-Aldrich); 0.01, 0.05, and 0.1 units of the enzyme were used for one million HL60 shcontrol and shHELLS cells and incubated for 5 min at 37 °C. The reaction was stopped with stop buffer (0.025 M EDTA, 0.5 M EGTA, 10% SDS, and 10 mg/ml proteinase K), followed by overnight incubation at 65 °C. The chromatin was subjected to phenol-chloroform (Himedia, MB078) extraction and precipitated at −80 °C, followed by washing with 100% and 70% ethanol. Chromatin digestion was assessed by electrophoresing the DNA on a 1.7% agarose gel. The band intensities of the MNase-digested ladder-like bands were calculated using the Gelanalyzer software v23.1.1.

### GSEA

Publicly available AML patients’ gene expression data from TCGA and the BeatAML cohort were utilized. For each data set, samples in the top and bottom 10 percentiles of HELLS expression were selected as “HELLS^high^” and “HELLS^low^” expression, respectively. Pathway analysis was conducted using GSEA, with Hallmark, Reactome, and KEGG pathways as reference, to investigate pathway differences between “HELLS^high^” and “HELLS^low^” samples.

### Transcriptomic data curation and bioinformatics analysis from BeatAML1 and BeatAML2

The gene expression and clinical metadata for the BeatAML1 (n = 672) and BeatAML2 (n = 942) cohorts were retrieved from cBioPortal. These cohorts were further checked for patients with *HELLS* gene expression, RPKM values ranging from 0.2 to 5.6, ELN-2017 risk stratification, blast percentages in PB (0–99.2 in BeatAML1 and BeatAML2) and bone marrow (0–97 in BeatAML1 and 3–98 in BeatAML2), and treatment response variables. After this stratification, the study cohort of AML patients was developed, with n = 451 and n = 671 for BeatAML1 and BeatAML2, respectively. Although the median RPKM value was 3.59 for HELLS expression in both BeatAML1 and BeatAML2 and in the AML patient cohort, we segregated patients into HELLSHigh (RPKM values 3–5.6) and HELLSLow (RPKM values 0.2–2.98) based on the rounded median RPKM. These patients were grouped into ELN-2017 risk categories: Favorable, Intermediate, and Adverse. Within each HELLS^High^ dataset from BeatAML1 and BeatAML2, the comparative analyses of immunophenotypic marker expression across hematopoietic precursors, myeloid lineage, and mature myeloid markers were performed and visualized using “ggplot2” (version 3.5.2) and “ggpubr” (version 0.6.0) in R Bioconductor for Adverse and Favorable risk patient groups. Survival outcomes and HRs for each ELN risk group in HELLS expression subgroup were estimated using Kaplan–Meier curves and Cox proportional hazard models in the “survival” (version 3.8.3), “survminer” (version 0.5.0), and “ggsurvfit” (version 1.1.0) packages in the R environment. Furthermore, the percentages of bone marrow and PB blasts, as well as clinical response rates, were also evaluated across these groups.

### Statistical analysis

Student’s *t* test (*p*-value < 0.05) was used for determining significance in the graphs, except when stated otherwise. Graphs and error bars reflect mean ± SD from the mean. Wilcoxon rank-sum tests were used for the bioinformatics analysis from BeatAML1 and BeatAML2 datasets. ELN-2017 risk category groups were compared and *p*-values < 0.05 were considered significant.

## Ethical considerations

The ethical permits for cord blood and AML patient samples (89/HEC/19 and 116/HEC/2022) were approved by the Institutional Ethical Committee (IEC)/Institutional Review Board (IRB) of the Institute of Life Sciences. Ethical permission for cord blood was obtained from the research committee (KIMS/PRC/12/2019) and IEC (KIIT/KIMS/IEC/41/2019) from the Kalinga Institute of Medical Sciences (KIMS). Ethical permission to use blood samples from AML patients for research purposes was obtained from the IEC of the Institute of Medical Sciences (IMS) and SUM Hospital, Siksha ‘O’ Anusandhan University (DMR/IMS.SH/SOA/180215 and IEC/IMS.SH/SOA/2023/480). Institutional biosafety committee (IBSC) approval (V-122-MISC/2007–08/01) was obtained for RNAi studies. All human studies reported in this manuscript were conducted in accordance with the principles embodied in the Declaration of Helsinki.

## Data availability

The dataset used in this study is curated from the following databases: BloodSpot (https://www.fobinf.com/), GEPIA2 (http://gepia2.cancer-pku.cn/), and cBioPortal (https://www.cbioportal.org/). The raw files for BeatAML1 and BeatAML2 were retrieved from cBioPortal in the myeloid subcategory. These files contained patient information and clinical profiles, which were further processed and analyzed using in-house scripts for data filtering and removal of missing information, as described in the manuscript.

## Supporting information

This article contains supporting information.

## Conflict of interest

The authors declare that they have no conflicts of interest with the contents of this article.

## References

[1] Geiman T.M., Durum S.K., Muegge K. (1998). Characterization of gene expression, genomic structure, and chromosomal localization of Hells (Lsh). Genomics.

[2] Jarvis C.D., Geiman T., Vila-Storm M.P., Osipovich O., Akella U., Candeias S. (1996). A novel putative helicase produced in early murine lymphocytes. Gene.

[3] Raabe E.H., Abdurrahman L., Behbehani G., Arceci R.J. (2001). An SNF2 factor involved in mammalian development and cellular proliferation. Dev. Dyn..

[4] Peixoto E., Khan A., Lewis Z.A., Contreras-Galindo R., Czaja W. (2022). The chromatin remodeler HELLS: a new regulator in DNA repair, genome maintenance, and cancer. Int. J. Mol. Sci..

[5] Chen X., Li Y., Rubio K., Deng B., Li Y., Tang Q. (2022). Lymphoid-specific helicase in epigenetics, DNA repair and cancer. Br. J. Cancer.

[6] Spruce C., Dlamini S., Ananda G., Bronkema N., Tian H., Paigen K. (2020). HELLS and PRDM9 form a pioneer complex to open chromatin at meiotic recombination hot spots. Genes Dev..

[7] Burrage J., Termanis A., Geissner A., Myant K., Gordon K., Stancheva I. (2012). The SNF2 family ATPase LSH promotes phosphorylation of H2AX and efficient repair of DNA double-strand breaks in mammalian cells. J. Cell Sci..

[8] Shinkai A., Hashimoto H., Shimura C., Fujimoto H., Fukuda K., Horikoshi N. (2024). The C-terminal 4CXXC-type zinc finger domain of CDCA7 recognizes hemimethylated DNA and modulates activities of chromatin remodeling enzyme HELLS. Nucleic Acids Res..

[9] Xu X., Ni K., He Y., Ren J., Sun C., Liu Y. (2021). The epigenetic regulator LSH maintains fork protection and genomic stability via MacroH2A deposition and RAD51 filament formation. Nat. Commun..

[10] Unoki M., Funabiki H., Velasco G., Francastel C., Sasaki H. (2019). CDCA7 and HELLS mutations undermine nonhomologous end joining in centromeric instability syndrome. J. Clin. Invest..

[11] Mallia S., Gambarelli G., Ciarrocchi A., Fragliasso V. (2025). HELLS: the transcriptional sentinel. Trends Cell Biol..

[12] Ni K., Ren J., Xu X., He Y., Finney R., Braun S.M.G. (2020). LSH mediates gene repression through macroH2A deposition. Nat. Commun..

[13] Ren J., Finney R., Ni K., Cam M., Muegge K. (2019). The chromatin remodeling protein Lsh alters nucleosome occupancy at putative enhancers and modulates binding of lineage specific transcription factors. Epigenetics.

[14] Liang X., Li L., Fan Y. (2022). Diagnostic, prognostic, and immunological roles of HELLS in pan-cancer: a bioinformatics analysis. Front. Immunol..

[15] Schuller S., Sieker J., Riemenschneider P., Kohler B., Drucker E., Weiler S.M.E. (2022). HELLS is negatively regulated by wild-type P53 in liver cancer by a mechanism involving P21 and FOXM1. Cancers (Basel).

[16] He C., Liu L. (2022). Hsa_circ_0072008 regulates cell proliferation, migration, and invasion in cervical squamous cell carcinoma via miR-1305/helicase, lymphoid specific (HELLS) axis. Bioengineered.

[17] Zhang G., Dong Z., Prager B.C., Kim L.J., Wu Q., Gimple R.C. (2019). Chromatin remodeler HELLS maintains glioma stem cells through E2F3 and MYC. JCI Insight.

[18] He X., Yan B., Liu S., Jia J., Lai W., Xin X. (2016). Chromatin remodeling factor LSH drives cancer progression by suppressing the activity of fumarate hydratase. Cancer Res..

[19] Wang R., Shi Y., Chen L., Jiang Y., Mao C., Yan B. (2015). The ratio of FoxA1 to FoxA2 in lung adenocarcinoma is regulated by LncRNA HOTAIR and chromatin remodeling factor LSH. Sci. Rep..

[20] Janus J.R., Laborde R.R., Greenberg A.J., Wang V.W., Wei W., Trier A. (2011). Linking expression of FOXM1, CEP55 and HELLS to tumorigenesis in oropharyngeal squamous cell carcinoma. Laryngoscope.

[21] Waseem A., Ali M., Odell E.W., Fortune F., Teh M.T. (2010). Downstream targets of FOXM1: CEP55 and HELLS are cancer progression markers of head and neck squamous cell carcinoma. Oral Oncol..

[22] Cousu C., Mulot E., De Smet A., Formichetti S., Lecoeuche D., Ren J. (2023). Germinal center output is sustained by HELLS-dependent DNA-methylation-maintenance in B cells. Nat. Commun..

[23] He Y., Ren J., Xu X., Ni K., Schwader A., Finney R. (2020). Lsh/HELLS is required for B lymphocyte development and immunoglobulin class switch recombination. Proc. Natl. Acad. Sci. U. S. A..

[24] Fan T., Schmidtmann A., Xi S., Briones V., Zhu H., Suh H.C. (2008). DNA hypomethylation caused by Lsh deletion promotes erythroleukemia development. Epigenetics.

[25] Lee D.W., Zhang K., Ning Z.Q., Raabe E.H., Tintner S., Wieland R. (2000). Proliferation-associated SNF2-like gene (PASG): a SNF2 family member altered in leukemia. Cancer Res..

[26] Prasad P., Ronnerblad M., Arner E., Itoh M., Kawaji H., Lassmann T. (2014). High-throughput transcription profiling identifies putative epigenetic regulators of hematopoiesis. Blood.

[27] Han Y., Ren J., Lee E., Xu X., Yu W., Muegge K. (2017). Lsh/HELLS regulates self-renewal/proliferation of neural stem/progenitor cells. Sci. Rep..

[28] De La Fuente R., Baumann C., Fan T., Schmidtmann A., Dobrinski I., Muegge K. (2006). Lsh is required for meiotic chromosome synapsis and retrotransposon silencing in female germ cells. Nat. Cell Biol..

[29] Basu J., Madhulika S., Murmu K.C., Mohanty S., Samal P., Das A. (2023). Molecular and epigenetic alterations in normal and malignant myelopoiesis in human leukemia 60 (HL60) promyelocytic cell line model. Front. Cell Dev. Biol..

[30] Bottomly D., Long N., Schultz A.R., Kurtz S.E., Tognon C.E., Johnson K. (2022). Integrative analysis of drug response and clinical outcome in acute myeloid leukemia. Cancer Cell.

[31] Tyner J.W., Tognon C.E., Bottomly D., Wilmot B., Kurtz S.E., Savage S.L. (2018). Functional genomic landscape of acute myeloid leukaemia. Nature.

[32] Cancer Genome Atlas Research N., Ley T.J., Miller C., Ding L., Raphael B.J., Mungall A.J. (2013). Genomic and epigenomic landscapes of adult de novo acute myeloid leukemia. N. Engl. J. Med..

[33] Hou X., Yang L., Wang K., Zhou Y., Li Q., Kong F. (2021). HELLS, a chromatin remodeler is highly expressed in pancreatic cancer and downregulation of it impairs tumor growth and sensitizes to cisplatin by reexpressing the tumor suppressor TGFBR3. Cancer Med..

[34] Law C.T., Wei L., Tsang F.H., Chan C.Y., Xu I.M., Lai R.K. (2019). HELLS regulates chromatin remodeling and epigenetic silencing of multiple tumor suppressor genes in human hepatocellular carcinoma. Hepatology.

[35] Yang Y., Zhang M., Wang Y. (2022). The roles of histone modifications in tumorigenesis and associated inhibitors in cancer therapy. J. Natl. Cancer Cent..

[36] Barral A., Dejardin J. (2023). The chromatin signatures of enhancers and their dynamic regulation. Nucleus.

[37] Dohner H., Estey E., Grimwade D., Amadori S., Appelbaum F.R., Buchner T. (2017). Diagnosis and management of AML in adults: 2017 ELN recommendations from an international expert panel. Blood.

[38] Shi Z.Y., Sun K., Li Z.Y., Xie D.H., Qin Y.Z. (2025). TIM-3 promotes proliferation of Acute Myeloid leukemia blasts. Biomedicines.

[39] Testa U., Pelosi E., Frankel A. (2014). CD 123 is a membrane biomarker and a therapeutic target in hematologic malignancies. Biomark Res..

[40] Akashi K., Nakao K., Minato N., Uemoto S. (2015). Innovative Medicine: Basic Research and Development.

[41] Yano M., Ouchida M., Shigematsu H., Tanaka N., Ichimura K., Kobayashi K. (2004). Tumor-specific exon creation of the HELLS/SMARCA6 gene in non-small cell lung cancer. Int. J. Cancer.

[42] Yang G., Fu J., Wang J., Ding M. (2025). HELLS knockdown inhibits the malignant progression of lung adenocarcinoma via blocking Akt/CREB pathway by downregulating KIF11. Mol. Biotechnol..

[43] Zocchi L., Mehta A., Wu S.C., Wu J., Gu Y., Wang J. (2020). Chromatin remodeling protein HELLS is critical for retinoblastoma tumor initiation and progression. Oncogenesis.

[44] Choi Y.J., Yoo N.J., Lee S.H. (2015). Mutation of HELLS, a chromatin remodeling gene, gastric and colorectal cancers. Pathol. Oncol. Res..

[45] Menon V., Gueble S.E. (2025). DNA repair helicases: from mechanistic understanding to therapeutic implications. NAR Cancer.

[46] Robinson M.H., Maximov V., Lallani S., Farooq H., Taylor M.D., Read R.D. (2019). Upregulation of the chromatin remodeler HELLS is mediated by YAP1 in Sonic Hedgehog Medulloblastoma. Sci. Rep..

[47] Xiao D., Huang J., Pan Y., Li H., Fu C., Mao C. (2017). Chromatin remodeling factor LSH is upregulated by the LRP6-GSK3beta-E2F1 axis linking reversely with survival in gliomas. Theranostics.

[48] Wong S.H., Goode D.L., Iwasaki M., Wei M.C., Kuo H.P., Zhu L. (2015). The H3K4-Methyl epigenome regulates leukemia stem cell oncogenic potential. Cancer Cell.

[49] Zhou R., Han L., Li G., Tong T. (2009). Senescence delay and repression of p16INK4a by Lsh via recruitment of histone deacetylases in human diploid fibroblasts. Nucleic Acids Res..

[50] Yu W., McIntosh C., Lister R., Zhu I., Han Y., Ren J. (2014). Genome-wide DNA methylation patterns in LSH mutant reveals de-repression of repeat elements and redundant epigenetic silencing pathways. Genome Res..

[51] Jeyaraju D.V., Alapa M., Polonskaia A., Risueno A., Subramanyam P., Anand A. (2024). Extended exposure to low doses of azacitidine induces differentiation of leukemic stem cells through activation of myeloperoxidase. Haematologica.

[52] Sherif H.A., Magdy A., Elshesheni H.A., Ramadan S.M., Rashed R.A. (2021). Treatment outcome of doxorubicin versus idarubicin in adult acute myeloid leukemia. Leuk. Res. Rep..

[53] Saha S., Samal P., Madhulika S., Murmu K.C., Chakraborty S., Basu J. (2022). SMARCD1 negatively regulates myeloid differentiation of leukemic cells via epigenetic mechanisms. Blood Adv..

