# Supplementary material for: Dysregulated HELLS expression alters cellular processes and serves as a potential prognostic marker in acute myeloid leukemia
Source: J Biol Chem. 2026 May 28;302(7):113210. doi: 10.1016/j.jbc.2026.113210 (PMC13310630; doi:10.1016/j.jbc.2026.113210)
Supplement: Supporting information [file mmc1.docx]

**Supporting Information**

**Dysregulated *HELLS* Expression alters cellular processes and serves as a potential prognostic marker in Acute Myeloid Leukemia**

Madhulika *et.al.*

1. **Supplementary Method**

IC_50_ values for 5-azacytidine (A2385, Sigma), cytarabine (C6645, Sigma), or doxorubicin (44583, Sigma) were determined. Briefly, 9×10^4^ HL-60 cells were treated with increasing (two-fold) concentrations of either 5-azacytidine (0–100 μM), cytarabine (0–100 μM), or doxorubicin (0–5 μM) in 900 μl for 48 h at 37 ⁰C in a CO_2_ incubator in a 12-well plate. After 48 h, 5 mg/mL of MTT (Himedia, MB186) at 1/10th of the original volume was added, and the mixture was further incubated for 3.5 h in the dark at 37⁰C in a CO_2_ incubator. After MTT incubation, 100 µl was transferred to a 96-well plate, an equal volume of DMSO was added, and the mixture was incubated for 10 minutes at room temperature. The absorbance was measured at 570 nm in a spectrophotometer. The absorbance values were analyzed in GraphPad Prism (Version 9) to determine IC_50_ values. The concentrations were taken on the x-axis and transformed to a log scale {X = log (X)}. The absorbance values for the different drug concentrations were then normalized to the highest (untreated) and lowest (highest drug concentration) values. Then, a nonlinear regression (log(inhibitor) vs. normalized response-variable slope) was used to fit the dose-response curve and calculate the IC_50_ value. Goodness-of-fit was determined from the regression model. Data represent mean ± SD.

1.
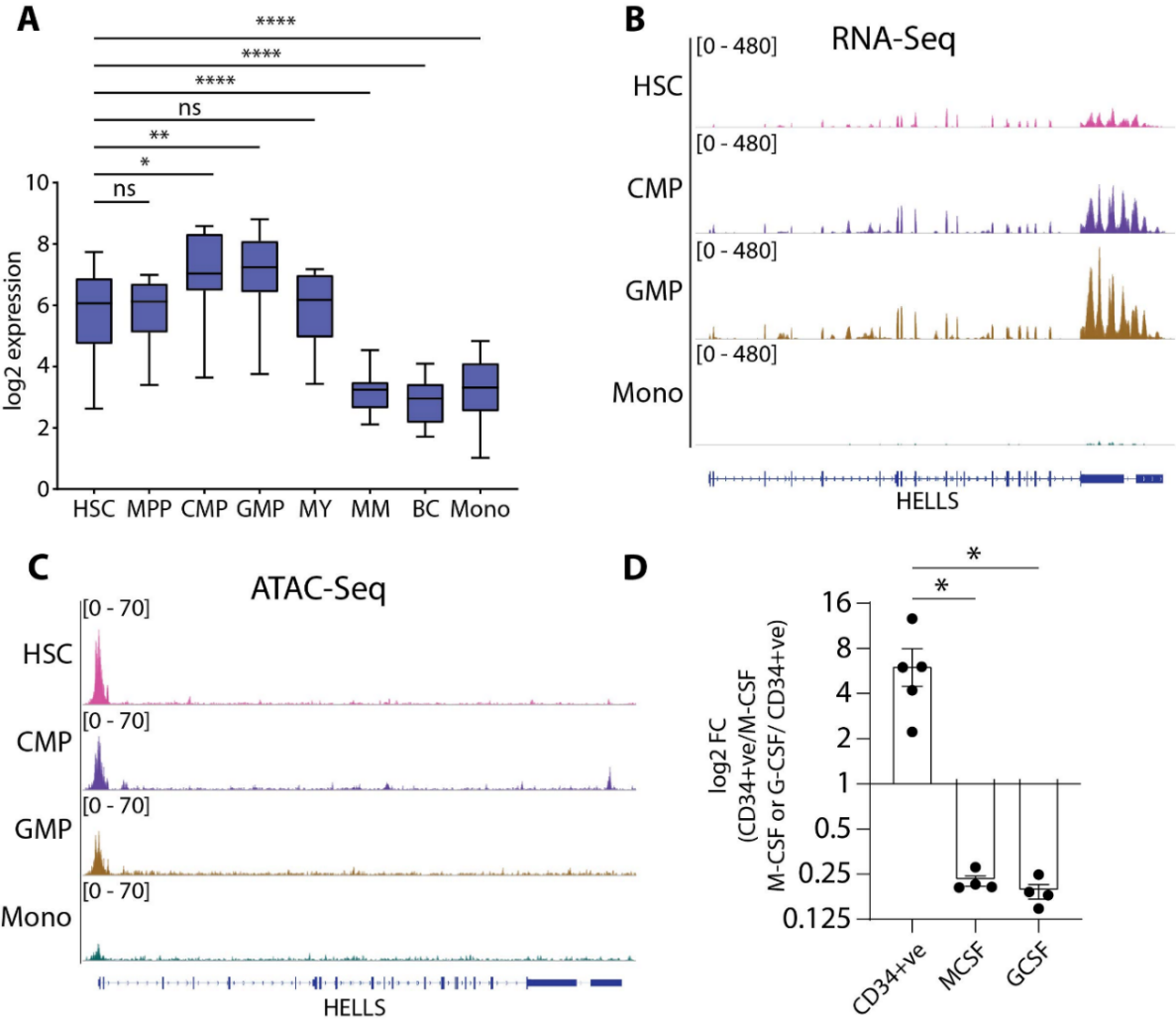
**Supporting Figures**

**Figure S1: Expression of *HELLS* in hematopoietic stem/progenitor is significantly reduced upon myeloid differentiation** (A) Log2 normalized count of *HELLS* expression from microarray data for hematopoietic stem cells (HSC), multipotent progenitors (MPP), common myeloid progenitor, (CMP), granulocyte monocyte progenitor (GMP), myelocyte (MY), metamyelocyte (MM), band cells (BC), and monocytes (Mono) from the BLOODSPOT (<https://www.fobinf.com/>) database. (B) Genome browser plot of RNA-sequencing peaks at gene bodies of *HELLS* across normal cells, HSC (n = 4), CMP (common myeloid precursors; n = 4), GMP (Granulocyte-monocyte precursors; n = 04), and monocytes (n = 4), respectively, from GSE74246. (C) Genome browser plot of ATAC-sequencing peaks at *HELLS* promoter region across normal cells, HSCs (n = 4), CMPs (n = 4), GMPs (n = 4), and monocytes (n = 4), respectively, from GSE74912. (D) Relative log2 fold change (CD34/M-CSF – 31; CD34/G-CSF – 32) of *HELLS* gene in cord-blood derived CD34+ cells (n = 5) compared with that in the M-CSF (macrophage colony-stimulating factor) (n = 4) (5.982 ± 1.981), and G-CSF (Granulocyte colony-stimulating factor) (n = 4) (6.015 ± 1.981) induced cells. Statistical parameter used – Unpaired t-test, ns - non-significant, * p-value < 0.05, ** p-value < 0.01, **** p-value <0.0001.


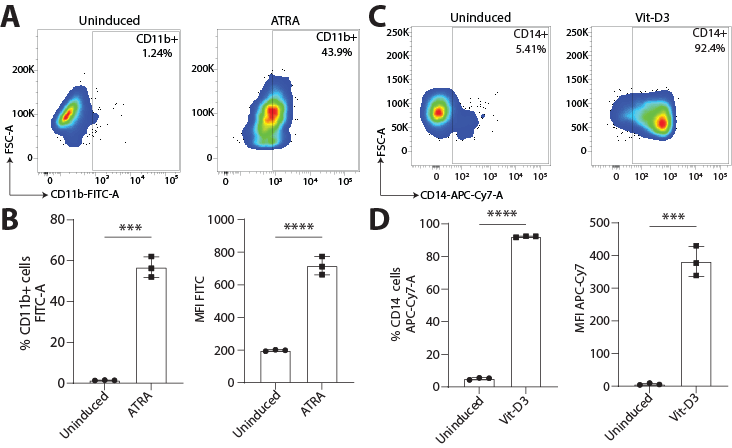


**Figure S2: Related to Figure 2. Loss of *HELLS* promotes myeloid differentiation.** HL-60 cells can be efficiently differentiated with ATRA and Vitamin D3 and therefore serve as an in vitro model of myeloid differentiation in this study. HL-60 cells were treated with 50 nM vitamin-D3 (Vit-D3) and 10 µM *all-trans* retinoic acid (ATRA) for 72 hours. (A-D) The representative pseudo-color plots generated by flow cytometry analysis of HL-60 cells induced with ATRA (SD = 55.41 ± 2.927) and Vitamin D3 (SD = 86.99 ± 0.4467) show enhanced expression of the myeloid differentiation markers CD11b (41-fold) and CD14 (17.8-fold), respectively, compared with uninduced cells. Bar plots generated from three biological replicates, from ATRA (A-B) and Vitamin D3 (C-D) inductions, show fold changes and median fluorescence intensity (MFI) of myeloid marker induction. Flow cytometry data were analyzed using FlowJo software version 10.9.0. All statistical parameters used in this figure are for n ≥ 3 independent experiments; error bars are mean ± S.E.M. ***p<0.001, ****p < 0.0001, two-tailed Student's t-test.


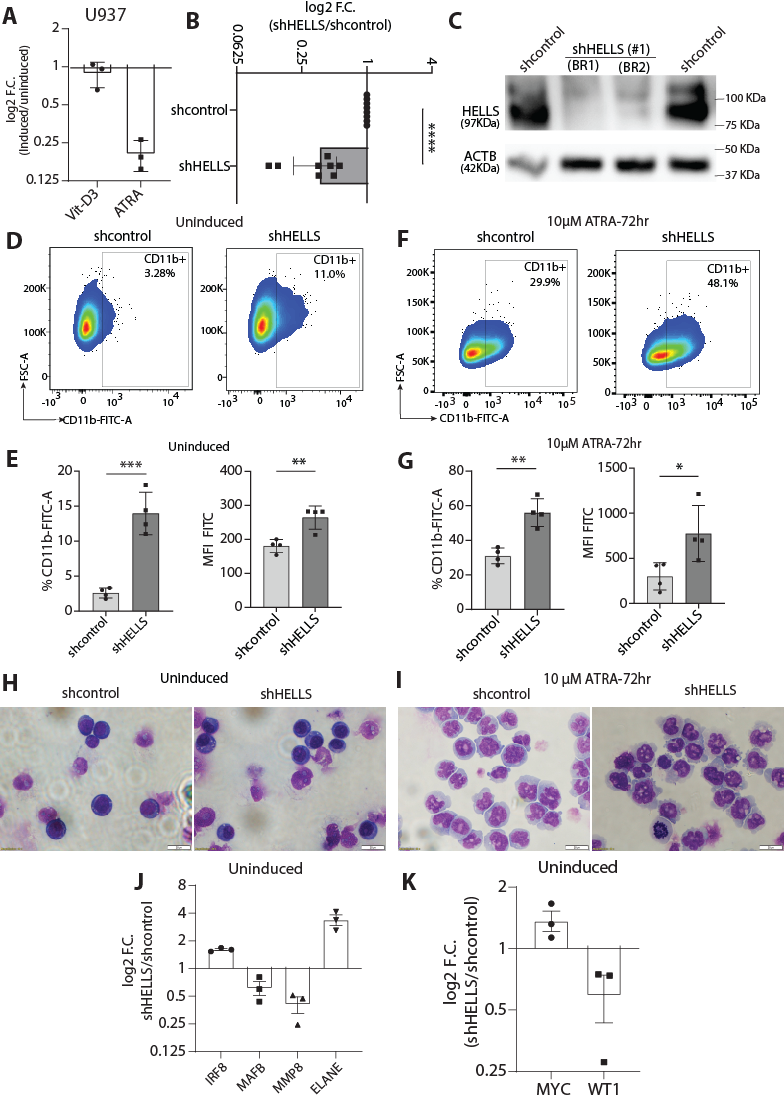


**Figure S3: Related to Figure 2.** **Loss of *HELLS* promotes myeloid differentiation** **in U937 promyelocytes.** (A) U937 cells were treated with 50 nM vitamin-D3 (Vit-D3) and 10 µM *all-trans* retinoic acid (ATRA) for 72 hours. Relative log2 fold change of HELLS gene expression in Vit-D3 and ATRA-treated U937 cells compared with uninduced U937 cells. Actin (*ACTB*) was used as a housekeeping control to determine relative fold change. (B-C) U937 cells were transduced with lentiviruses containing either an empty vector (shcontrol) or shRNA targeting HELLS (shHELLS). Transduced cells were selected with puromycin for 96 hours prior to RNA and protein isolation. *HELLS* knockdown was confirmed by qPCR analysis (B) using *ACTB* as a housekeeping control gene. Total protein cell lysates (60 µg) from shcontrol and shHELLS were used for immunoblotting to detect HELLS and ACTB as a loading control. Representative immunoblot image for U937 (C). (D-G) Flow cytometry analysis of U937 cells with shcontrol or shHELLS showing myeloid differentiation marker CD11b in uninduced (D-E) and 10 µM ATRA (F-G) for 72 hours ATRA induction. Representative pseudo-color plots with grids, showing percent positive for CD11b-FITC populations. MFI plots of shcontrol and shHELLS U937 cells. (H-I) Representative Giemsa-stained images of shcontrol and shHELLS U937 cells in uninduced (H) and 10 µM for 72 hours (I). Relative log2 fold change of (J) differentiation-related genes, (*IRF8* (0.7452 ± 0.0430), *MAFB* (0.1849 ± 0.1067), *MMP8* (0.1439 ± 0.0830), *ELANE* (0.7794 ± 0.450) (K) leukemic transcription factors, (*WT1* (0.2679 ± 0.1547), *MYC* (0.2679 ± 0.1547)) in shHELLS U937 cells compared with shcontrol U937 cells. Flow cytometry data were analyzed using FlowJo software version 10.9.0. All statistical parameters used in this figure for n ≥ 3 independent experiments; error bars are mean ± S.E.M. ns - non-significant, *p < 0.05, **p < 0.01, ***p<0.001, ****p < 0.0001, two-tailed Student's t-test.


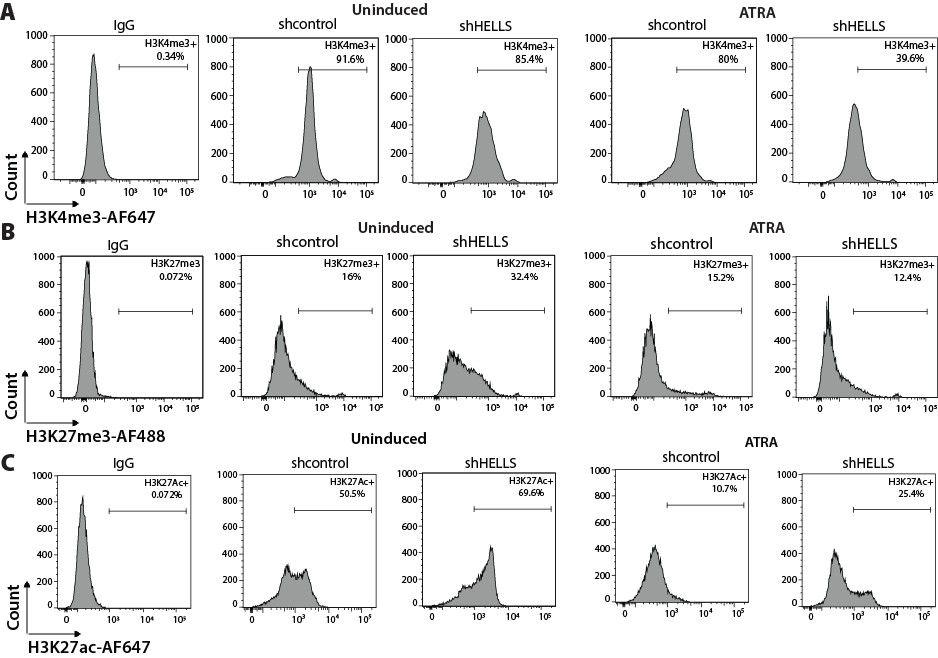


**Figure S4. Related to Figure 5.** **Loss of *HELLS* alters global histone modifications.** (A) Flow cytometry analysis of HL-60 cells with shcontrol or shHELLS showing active histone marks H3K4me3 in uninduced and with 1 µM ATRA induction for 48 hours. Representative histogram plots with grids, showing percent positive population for CD11b-FITC populations. (B) Flow cytometry analysis of HL-60 cells with shcontrol or shHELLS showing repressive histone mark H3K27me3 in uninduced and 1 µM ATRA for 48 hours. Representative histogram plots with grids, showing percent positive population for CD11b-FITC. (C) Flow cytometry analysis of HL-60 cells with shcontrol or shHELLS showing active enhancer histone mark H3K27ac on active enhancers in uninduced and with 1 µM ATRA induction for 48 hours. Representative histogram plots with grids, showing percent positive population for CD11b-FITC. IgG for the respective fluorophore is taken as a negative control for each histone modification tested. Flow cytometry data were analyzed using FlowJo software version 10.9.0.


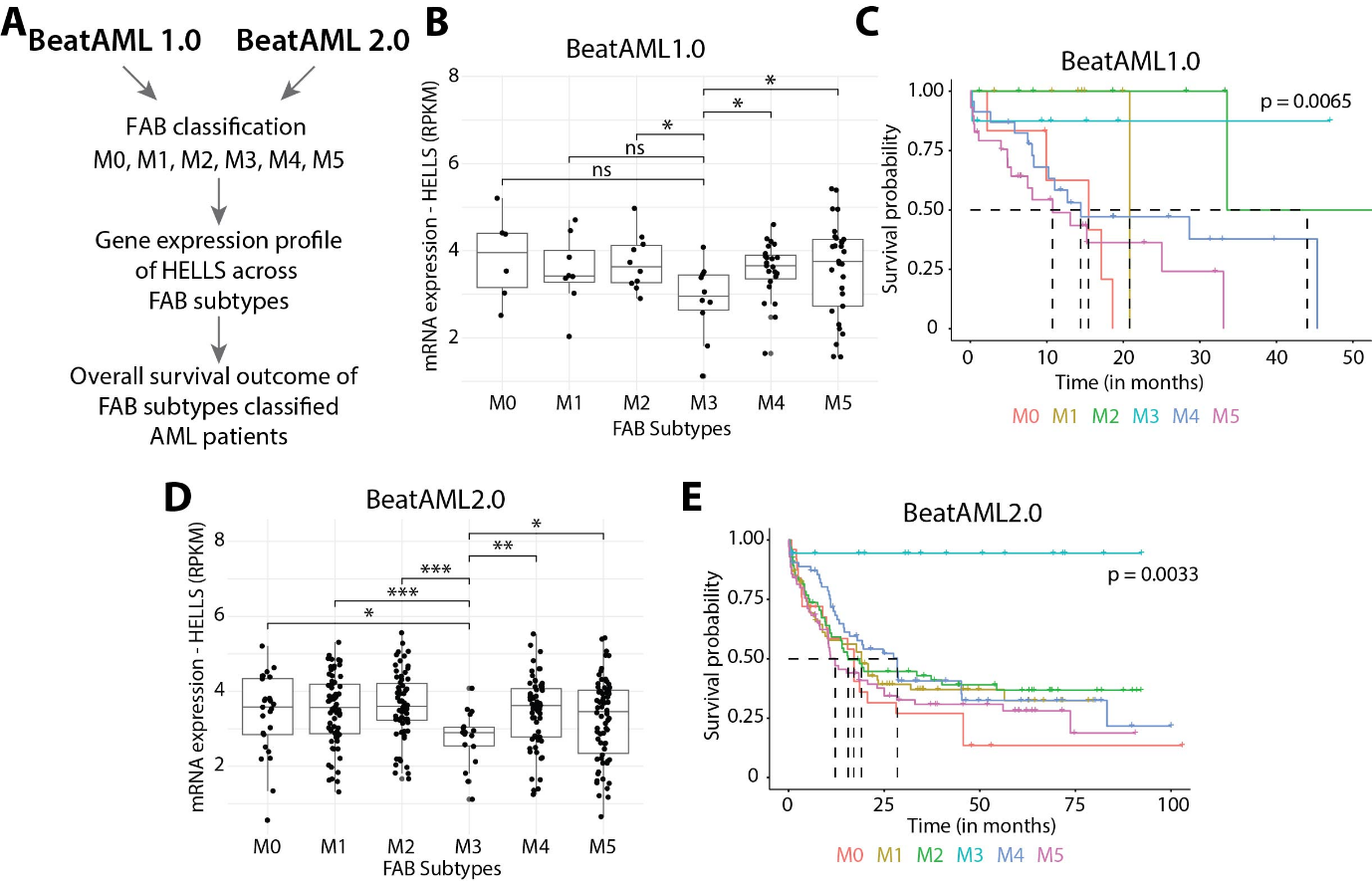


**Figure S5. Related to Figure 6**. **M3 subtype has the lowest expression of *HELLS* compared with other FAB subtypes.** (A) Schematic representation of *HELLS* expression data curated from cBioportal for BeatAML1.0 and BeatAML2.0, followed by classifying the AML patients in different FAB subtypes and analyzing their overall survival. (B and D) Box plot showing *HELLS* mRNA expression across FAB subtypes in BeatAML1.0 and BeatAML2.0. (C and E) Overall survival determined by the Kaplan-Meier plot of patients based on FAB Classification with risk categories of M0, M1, M2, M3, M4, M5 in BeatAML1.0 and BeatAML2.0. Statistical analysis by Wilcoxon test: ns - non-significant, *p < 0.05, **p < 0.01, ***p<0.001.


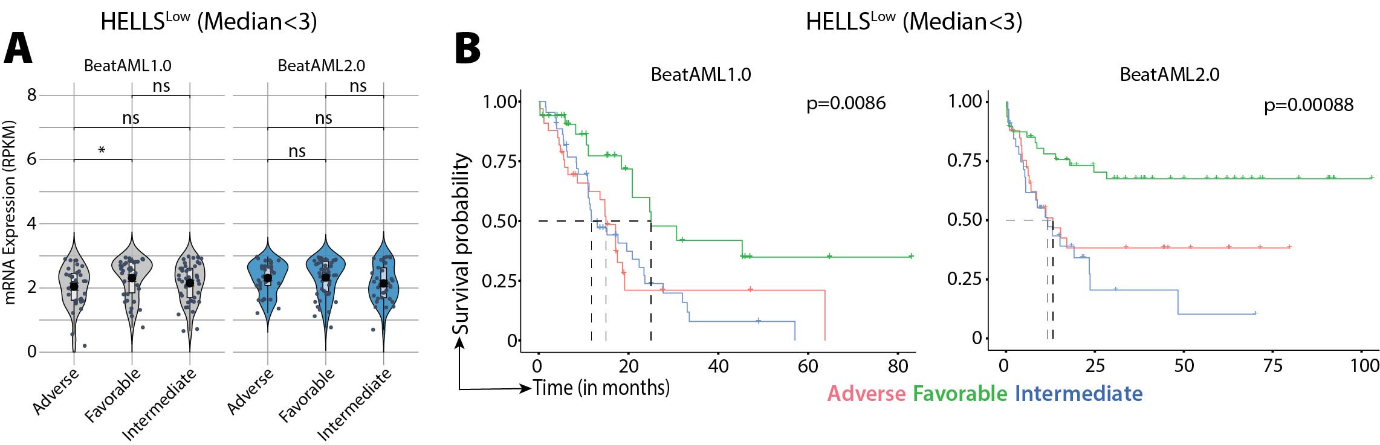


**Figure S6. Related to Figure 6.** **High gene expression of *HELLS* is a potential indicator of poor prognosis.** (A) Violin Plots showing variation of HELLS^Low^ cohort expression in Adverse, Favorable, and Intermediate Risk groups in BeatAML1.0 and BeatAML2.0 datasets as per ELN-2017. (B) Overall survival curves from the Kaplan-Meier plot for the ELN-2017 Risk Classification in the HELLS^Low^ cohort, with corresponding median survival. Statistical analysis by Wilcoxon test: ns - non-significant, *p < 0.05.


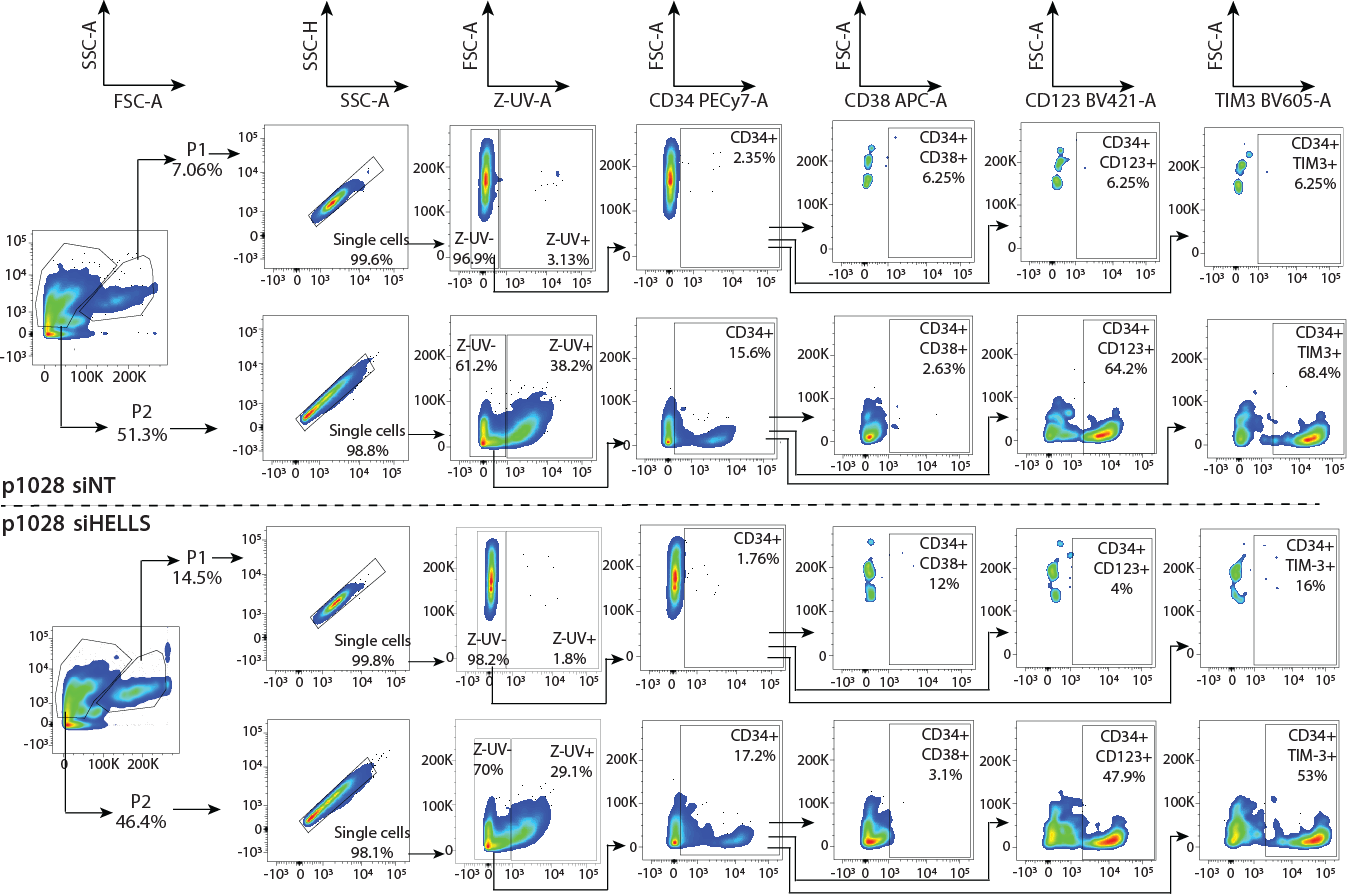


**Figure S7. Related to Figure 7. Loss of *HELLS* in patient-derived CD34+ LSPCs reduces leukemic burden and increases apoptosis.** An ex vivo model was used in this study, in which bone marrow aspirates from patients with AML were employed. Mononuclear cells were isolated by density gradient centrifugation, cultured, and then transfected with siNT or siHELLS. The figure represents a flow cytometry analysis pipeline for representative patient-derived LSPCs transfected with siNT or siHELLS. Forward and side scatter (FSC-A vs SSC-A) revealed two cell populations, P1 and P2, in all transfected samples, which were gated separately. Therefore, we analyzed the two populations separately to determine the percentage and other cellular parameters. After doublet discrimination, live cells (Zombie UV-negative) were analyzed for CD34+ cells. CD38+, CD123+, and TIM-3+ populations were determined in each P1 and P2 population. The P1 population represented a minor cellular event compared with the P2 population; therefore, the analysis shown in Figure 7 was performed on the P2 population. FMOs and respective IgG controls for each antibody (CD34-PE Cy7, CD38-APC, CD123-BV421, TIM-3-BV605) were taken as a control. ‘p’ in p1028 stands for AML patient. Flow cytometry data were analyzed using FlowJo software version 10.10.0.


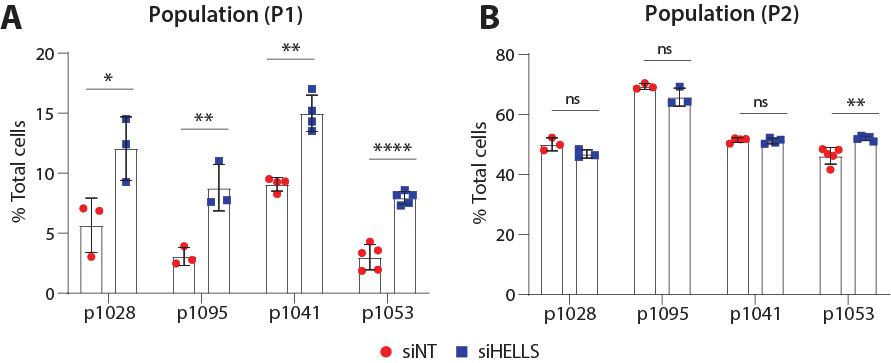


**Figure S8. Related to Figure 7. Loss of *HELLS* in patient-derived CD34+ LSPCs reduces leukemic burden and increases apoptosis.** This figure shows the percentage of total cells in each P1 and P2 population, as described in Figure S7. It presents the range of cell percentages for each AML patient in this study. (A-B) Bar graphs showing the percentage of P1 (A) and P2 (B) populations across different patient samples as analyzed by flow cytometry post-transfection (siNT in red and siHELLS in blue). Different numbers of independent transfections per patient were performed, and transfections with significant HELLS knockdown were selected for analysis. All statistical parameters used in this figure for n ≥ 3 independent experiments; error bars are mean ± S.E.M. ns - non-significant, *p< 0.05, **p<0.01, ***p<0.001, two-tailed Student's t-test.


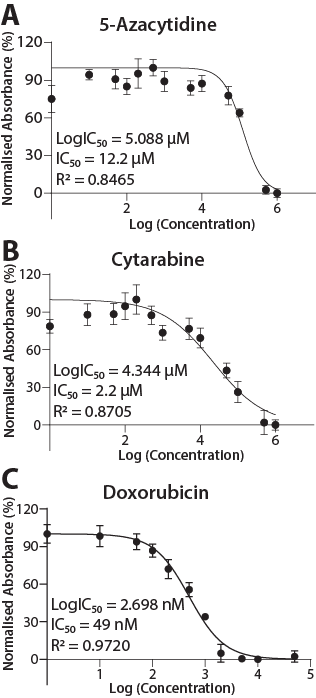


**Figure S9. Related to Figure 8. Half-maximal inhibitory concentration for 5-azacytidine, cytarabine, and doxorubicin in HL-60 cells.** (A-C) HL-60 cells were treated with increasing concentrations of either (A) 5-azacytidine (0–100 μM), (B) cytarabine (0–100 μM) or (C) doxorubicin (0–5 μM) for 48 h, and cell viability was assessed by MTT assay. Data represent mean ± SD (n = 7 technical repeats for each concentration). Nonlinear regression [log(inhibitor) vs. normalized response - variable slope] was used to fit the dose-response curve and to calculate the IC_50_ value. Goodness-of-fit was determined from the regression model. The graphs were plotted using the GraphPad Prism version 9.0.


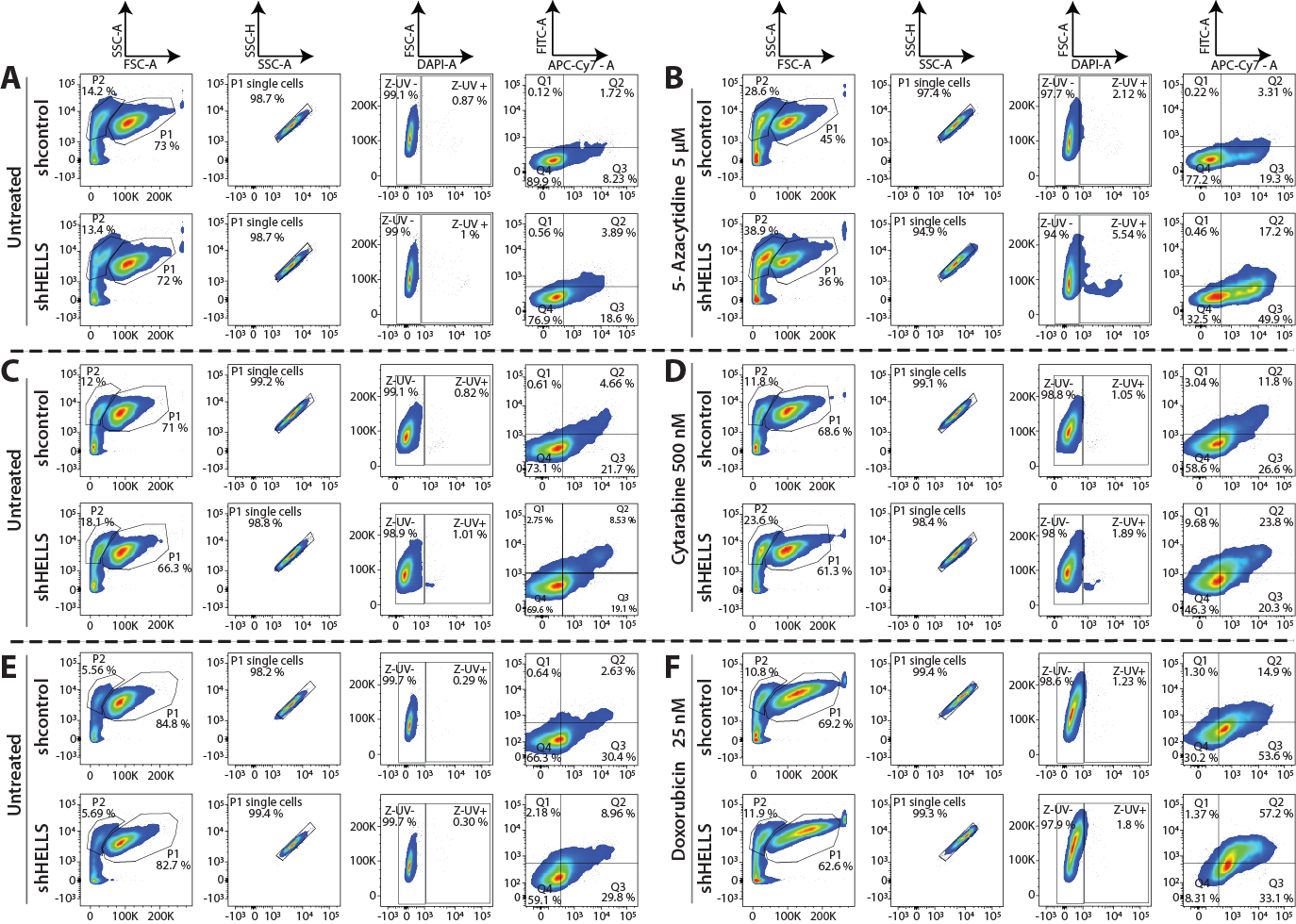


**Figure S10. Related to Figure 8. *HELLS* depletion enhances drug sensitivity in leukemic cells.** HL-60 cells were treated with varying concentrations of FDA-approved drugs (below IC_50_) to assess drug sensitivity in the absence of HELLS, as measured by flow cytometry. The drug concentrations used in the experiments had > 95% live cells (P1 population). Representative flow cytometry data for the highest concentration of 5-azacytidine (5 µM), (A-B), cytarabine (500 nM) (C-D), and doxorubicin (50 nM) (E-F) are shown. The untreated shcontrol cells (left) and drug-treated shHELLS (right) are shown. The data analysis steps for the P1 population, based on the scatter plots (FSC-A vs SSC-A) for each drug, are as follows: doublet discrimination, live cell selection (Zombie UV-negative), and analysis of myeloid differentiation markers CD11b+ and CD14+ (double-positive, Q2 quadrant). Representative pseudo-color plots with gating strategy and analysis for negative and positive populations for double-stained CD11b and CD14 populations. Flow cytometry data were analyzed using FlowJo software version 10.10.0.

1. **Supporting Tables**

**Table S1:** Summary of AML patient information from BeatAML1.0 and BeatAML2.0

| **Category** | **Sub-category** | **BeatAML1** | **BeatAML2** |
| --- | --- | --- | --- |
| **Number of samples** | Total HELLS cohort | 451 | 671 |
|  | HELLS^High^ cohort | 328 | 476 |
|  | HELLS^Low^ cohort | 123 | 195 |
| **Sex** | Number of males | 258 | 381 |
|  | Number of females | 193 | 290 |
| **Age Group** | Median Age | 61 | 61 |
| **Blast % - Bone Marrow** | Total (Range) | 0-97 | 3-98 |
|  | HELLS^High^ - Adverse | 60 (3-96) | 75 (3-90) |
|  | HELLS^High^ - Favorable | 72 (2-97) | 70 (18-97) |
|  | HELLS^High^ - Intermediate | 61.25 (0-95) | 81 (9-95) |
|  | HELLS^Low^ - Adverse | 70 (6-98) | 61 (3-98) |
|  | HELLS^Low^ - Favorable | 80 (1.5-91.8) | 85 (1.5-95) |
|  | HELLS^Low^ - Intermediate | 70 (0.4-90) | 78 (15-96) |
| **Blast % - Peripheral blood** | HELLS^High^ - Adverse | 41 (0-99.2) | 45.9 (0-99.2) |
|  | HELLS^High^ - Favorable | 48 (0-97) | 56.5 (0-96) |
|  | HELLS^High^ - Intermediate | 38 (0-99) | 60 (2-94) |
|  | HELLS^Low^ - Adverse | 58.5 (0-94) | 77 (0-96) |
|  | HELLS^Low^ - Favorable | 32(0-95) | 60 (0-97) |
| **Survival Time (in months)** | HELLS^Low^ – Intermediate  HELLS^High^ - Adverse  HELLS^High^ - Favorable  HELLS^High^ -Intermediate  HELLS^Low^ - Adverse  HELLS^Low^ - Favorable  HELLS^Low^ - Intermediate | 60.5 (0-92)  10.5  28.6  12.7  15  25  11.7 | 77 (7-88)  8.05  NA  9.47  13.2  NA  11.7 |
| **Hazard Ratio (ELN)** | HELLS^High^ - Adverse | 1 | 1 |
|  | HELLS^High^ - Favorable | 0.37 | 0.31 |
|  | HELLS^High^ - Intermediate | 0.73 | 0.78 |
|  | HELLS^Low^ - Adverse | 1 | 1 |
|  | HELLS^Low^ - Favorable | 0.39 | 0.40 |
| **Statistics** |  |  |  |
| **Blast% in Bone Marrow** | Adverse vs. Favorable | 0.01 | 0.89 |
| (p-values) | Favorable vs. Intermediate | 0.04 | 0.10 |
| **Blast% in Peripheral Blood** | Adverse vs. Favorable | 0.22 | 0.45 |
| (p-values) | Favorable vs. Intermediate | 0.19 | 0.76 |
| **Median RPKM Value** | HELLS^High^ - Adverse | 3.99 | 4.01 |
|  | HELLS^High^ - Favorable | 3.63 | 3.75 |
|  | HELLS^High^ - Intermediate | 3.96 | 3.93 |
|  | HELLS^Low^ - Adverse | 2.2 | 2.37 |
|  | HELLS^Low^ - Favorable | 2.56 | 2.48 |
|  | HELLS^Low^ – Intermediate | 2.24 | 2.09 |
| **FAB subtype** | M0 | 3.96 | 3.58 |
|  | M1 | 3.42 | 3.57 |
|  | M2 | 3.63 | 3.6 |
|  | M3 | 2.96 | 2.9 |
|  | M4 | 3.66 | 3.62 |
|  | M5 | 3.76 | 3.46 |
| **p- values** | M3 vs M0 | 0.17 | 0.02 |
|  | M3 vs M1 | 0.12 | 0.0008 |
|  | M3 vs M2 | 0.02 | 0.00016 |
|  | M3 vs M4 | 0.04 | 0.0018 |
|  | M3 vs M5 | 0.05 | 0.027 |
| **FAB subtype** (Median survival) | M0 | 15.5 | 17.1 |
|  | M1 | 20.8 | 19.1 |
|  | M2 | 44.0 | 15.5 |
|  | M3 | NA | NA |
|  | M4 | 14.4 | 28.4 |
|  | M5 | 10.8 | 12.2 |
| **Hazard Ratio (FAB)** | HELLS^Total^ - M0 | 1 | 1 |
|  | HELLS^Total^ - M1 | 0.16 | 0.74 |
|  | HELLS^Total^ - M2 | 0.07 | 0.65 |
|  | HELLS^Total^ - M3 | 0.12 | 0.05 |
|  | HELLS^Total^ - M4 | 0.52 | 0.63 |
|  | HELLS^Total^ - M5 | 0.94 | 0.88 |

**Table S2:** Summary of AML patient metadata used for ex vivo experiments

| **Category** | **Parameter** | **p1028** | **p1041** | **p1053** | **p1095** |
| --- | --- | --- | --- | --- | --- |
| **Clinical** | **Age (years)** | 47 | 58 | 25 | 44 |
|  | **Sex** | Female | Male | Female | Female |
|  | **BM Blasts (%)** | 29 | 11 | 35 | 61 |
|  | **Platelet Count (×10³/µL)** | 44 | 165 | 275 | 20 |
|  | **Neutrophils (%)** | 17 | 52 | 71.8 | 34 |
| **Markers** | **cyMPO** | Positive | Positive | Positive | Positive |
|  | **CD33** | Positive | Positive | Positive | Positive |
|  | **CD13** | Positive | Positive | Positive | Positive |
|  | **CD117** | NA* | Positive | Positive | Negative |
|  | **CD11c** | Positive | NA* | Positive | NA* |
|  | **CD64** | Positive | Negative | Positive | Positive |
|  | **CD14** | Positive | Positive | NA* | Negative |
|  | **CD15** | Positive | Negative | NA* | NA* |
|  | **CD36** | Positive | Negative | NA* | Negative |
|  | **CD34** | Negative | Negative | Positive | Positive |
|  | **HLA-DR** | Positive | Positive | Positive | Positive |
|  | **CD19** | Negative | Negative | NA* | Negative |
|  | **CD7** | Negative | Positive | NA* | Negative |

*NA- Not available

**Table S3.** List of antibodies used in this study

| Sl. No | Name | Company | Catalog No. | Lot No. | Application |
| --- | --- | --- | --- | --- | --- |
| 1 | HELLS | Invitrogen | PA564099 | SJ2464238A | WB |
| 2 | PTEN | CST | 9559S | 19 | WB |
| 3 | CDKN1A | ABM | A1483-20ul | 3560229104 | WB |
| 4 | PARP | CST | 9542T | 15 | WB |
| 5 | Cleaved PARP | CST | 5625T | 13 | WB |
| 6 | Actin | GeneTex | GTX629630 | 41554 | WB |
| 7 | CD11b-FITC | BD Biosciences | 558123 | 88617 | FC |
| 8 | AnnexinV-FITC | BD Biosciences | 556419 | 7209930 | FC |
| 9. | H3K4me3-AF647 | CST | 12064S | 5 | FC |
| 10 | H3K27me3-AF488 | CST | 5499S | 9 | FC |
| 11 | H3K27ac-AF647 | CST | 39030S | 2 | FC |
| 12 | MPO | ABM | A1374-20ul | 5500002208 | IF |
| 13 | PI | HIMEDIA | TC252 |  | FC & Comet |
| 14 | Anti-rabbit-AF568 | Invitrogen | A-11036 | 1924788 | IF |
| 15 | CD34 - PE Cy7 | BD Biosciences | 560710 | 7341871, 3199337 | FC |
| 16 | CD38 - APC | BD Biosciences | 560980 | 8180845 | FC |
| 17 | CD123 – BV421 | BD Biosciences | 563362 | 3165881 | FC |
| 18 | TIM-3 – BV605 | BD Biosciences | 742856 | 9072562 | FC |
| 19 | Zombie UV | Biolegend | B342952 | B342952 | FC |
| 20 | CD14 – APC Cy7 | Tonbo Biosciences | 25-0149-T100 | C0149110222253 | FC |
| 21 | Anti-mouse HRP-IgG | CST | 7076P2 | 32 | WB |
| 22 | Anti-rabbit HRP-IgG | CST | 7074P2 | 28 | WB |

WB = Western blotting; FC = Flow Cytometry; IF = Immunofluorescence

**Table S4.** Oligonucleotide details used in this study

| **Sl.no** | **Name** | **Forward Primer (5’-3’)** | **Reverse Primer (5’-3’)** |
| --- | --- | --- | --- |
| 1. | *HELLS* | ATGCACAGCTTCAACACGGA | ATCCGACTGGGGGTTCCAAT |
| 2. | *ACTIN* | TCTCCATGTCGTCCCAGTTG | ATTCCTATGTGGGCGACGAG |
| 3. | *IRF8* | ATGTGTGACCGGAATGGTGG | AGTCCTGGATACATGCTACTGTC |
| 4. | *MAFB* | AGCAAGCTGAGGGTCTTTGT | TGATGCAAAATGCCCGGAAC |
| 5. | *MYC* | CCCTCCACTCGGAAGGACTA | GCTGGTGCATTTTCGGTTGT |
| 6. | *WT1* | TGCTTACCCAGGCTGCAATAA | TTCTCACCAGTGTGCTTCCTG |
| 7. | *MMP8* | CCGAAGAAACATGGACCAACACCTC | TGAGCGAGCCCCAAAGAAT |
| 8. | *ELANE* | CGTGGCGAATGTAAACGTCC | TTTTCGAAGATGCGCTGCAC |
| 9. | *PTEN* | TGAGTTCCCTCAGCCGTTACCT | GAGGTTTCCTCTGGTCCTGGTA |
| 10. | *CDKN1A* | TGTCCGTCAGAACCCATGC | AAAGTCGAAGTTCCATCGCTC |
| 11. | *CDKN1B* | TAATTGGGGCTCCGGCTAACT | TGCAGGTCGCTTCCTTATTCC |
| 12. | *CDKN2AIP* | CCTGCCGTTGTTACCTGAGAG | GTGTATAGGGTCGGCCATCAA |
| 13. | *CDKN2C* | GGGGACCTAGAGCAACTTACT | CAGCGCAGTCCTTCCAAAT |
| 14. | *CDKN2D* | AGTCCAGTCCATGACGCAG | ATCAGGCACGTTGACATCAGC |

.
